# Effects of Reduced-Dose Anti-Human T-Lymphocyte Globulin on Overall and Donor-Specific T-Cell Repertoire Reconstitution in Sensitized Kidney Transplant Recipients

**DOI:** 10.3389/fimmu.2022.843452

**Published:** 2022-02-25

**Authors:** Constantin Aschauer, Kira Jelencsics, Karin Hu, Mariella Gregorich, Roman Reindl-Schwaighofer, Sabine Wenda, Thomas Wekerle, Andreas Heinzel, Rainer Oberbauer

**Affiliations:** ^1^ Division of Nephrology and Dialysis, Department of Medicine III, Medical University of Vienna, Vienna, Austria; ^2^ Section for Clinical Biometrics, Center for Medical Statistics, Informatics and Intelligent Systems, Medical University of Vienna, Vienna, Austria; ^3^ Department of Blood Group Serology and Transfusion Medicine, Medical University Vienna, Vienna, Austria; ^4^ Division of Transplantation, Department of General Surgery, Medical University of Vienna, Vienna, Austria

**Keywords:** kidney transplantation, immune reconstitution, anti-T lymphocyte globulin, allorepertoire, T-cell receptor (TCR) repertoire, lymphocyte depletion therapy, alloreactivity

## Abstract

**Background:**

Pre-sensitized kidney transplant recipients have a higher risk for rejection following kidney transplantation and therefore receive lymphodepletional induction therapy with anti-human T-lymphocyte globulin (ATLG) whereas non-sensitized patients are induced in many centers with basiliximab. The time course of lymphocyte reconstitution with regard to the overall and donor-reactive T-cell receptor (TCR) specificity remains elusive.

**Methods/Design:**

Five kidney transplant recipients receiving a 1.5-mg/kg ATLG induction therapy over 7 days and five patients with 2 × 20 mg basiliximab induction therapy were longitudinally monitored. Peripheral mononuclear cells were sampled pre-transplant and within 1, 3, and 12 months after transplantation, and their overall and donor-reactive TCRs were determined by next-generation sequencing of the TCR beta CDR3 region. Overall TCR repertoire diversity, turnover, and donor specificity were assessed at all timepoints.

**Results:**

We observed an increase in the donor-reactive TCR repertoire after transplantation in patients, independent of lymphocyte counts or induction therapy. Donor-reactive CD4 T-cell frequency in the ATLG group increased from 1.14% + -0.63 to 2.03% + -1.09 and from 0.93% + -0.63 to 1.82% + -1.17 in the basiliximab group in the first month. Diversity measurements of the entire T-cell repertoire and repertoire turnover showed no statistical difference between the two induction therapies. The difference in mean clonality between groups was 0.03 and 0.07 pre-transplant in the CD4 and CD8 fractions, respectively, and was not different over time (CD4: F(1.45, 11.6) = 0.64 p = 0.496; CD8: F(3, 24) = 0.60 p = 0.620). The mean difference in R20, a metric for immune dominance, between groups was -0.006 in CD4 and 0.001 in CD8 T-cells and not statistically different between the groups and subsequent timepoints (CD4: F(3, 24) = 0.85 p = 0.479; CD8: F(1.19, 9.52) = 0.79 p = 0.418).

**Conclusion:**

Reduced-dose ATLG induction therapy led to an initial lymphodepletion followed by an increase in the percentage of donor-reactive T-cells after transplantation similar to basiliximab induction therapy. Furthermore, reduced-dose ATLG did not change the overall TCR repertoire in terms of a narrowed or skewed TCR repertoire after immune reconstitution, comparable to non-depletional induction therapy.

## Background

Prevention of rejection and achievement long-lasting graft function represents one of the major challenges after kidney transplantation. Especially highly immunized individuals are at a higher risk for alloimmunity, and therefore medical immunosuppression is increased to eradicate preexisting donor-reactive lymphocytes and reduce donor-specific anti-human leukocyte antigen (HLA) antibodies (DSAs) in these recipients (1–3).

Patients at risk receive a lymphodepletional induction therapy in most transplant centers such as rabbit anti-human T-lymphocyte globulin (ATLG) or another lymphodepletional agent combined with plasma exchange or immunoadsorption (3).

Dosing for ATLG is heterogeneous in clinical practice as high dosing leads to a profound long-lasting lymphopenia with functional impaired immune cells following reconstitution and increases the incidence of infections and cancer (4, 5). Therefore, lower dosing regimens have been evaluated to reduce side effects without increased risk of rejecting the allograft (6–8).

ATLG provides multifaceted immunomodulation, including T-cell depletion in blood and peripheral lymphoid tissues, induction of apoptosis in B-cell lineages, interference with dendritic cell functional properties, induction of regulatory T-cells and NKT cells, and skewed immune repertoire and impaired T-cell function after reconstitution (9–11).

Before the upcoming of new techniques and tools to define donor-specific T-cells, studies have been focusing mostly on the phenotype and functionality but to a lesser extent on the specificity of the reconstituted T-cells (5, 11–18).

Cherkasky et al. first observed a marked and prolonged donor hyporesponsiveness with minimal effects on non-donor responses after Thymoglobuline^®^ (ATG) induction using ELISPOT assays of the reconstituted T-cell repertoire (19). The underlying mechanisms for this donor hyporesponsiveness were not elucidated, but a promotion of regulatory T-cells that are specific for donor antigens following lymphodepletion have been hypothesized.

These findings support the hypothesis that the ATG effect is the result not only from immunodepletion but also from the induction of T-cells that control allogeneic immune responses.

Improvements in next-generation sequencing (NGS) technologies have made it possible to characterize the overall and donor-reactive T-cell repertoire by T-cell receptor (TCR) sequencing of the circulating T-cells (20, 21). In addition, it is possible to specifically define the donor-reactive TCR repertoire pre-transplant and to subsequently track the donor-reactive TCRs after transplantation (22–25).

Recently, we were able to prove the enrichment of these predefined donor-reactive T-cells not only in the circulating TCR repertoire but also in the rejecting kidney allograft, underlining the immunological importance of these cells in non-sensitized kidney transplant recipients (23).

In this study, we sought to describe the effects of reduced-dose lymphodepletional therapy compared to basiliximab induction therapy and the reconstitution of the overall and donor-reactive TCR repertoire in kidney transplant recipients. We aimed not only to characterize lymphocyte reconstitution on a phenotype level but also to describe the TCR repertoire in a reduced-dose ATLG regimen making it possible to evaluate shifts in the shape of the immune repertoire and to also detect the reappearance of donor-reactive T-cells following reduced lymphodepletional induction therapy.

## Methods/Design

### Subjects

Five pre-sensitized kidney transplant recipients, defined as the presence of suspected DSAs and five recipients without preformed DSAs, were included prior to transplantation. The included subjects did not experience a cellular rejection episode within the observation time. However, patient R294 in the ATLG group required hospital admission for urinary tract infection (at 1.5 and 4 months post-transplant) and COVID-19 infection (at 6 months post-transplant) during the follow-up period. Sampling timepoints for this patient did not coincide with hospital admissions. Pre-transplant DSA positivity was defined as a mean fluorescence intensity (MFI) of >2,000. With the exception of patient R192 who experienced a reoccurrence of a preformed DSA at high titers, only low-titer DSAs or no DSAs became detectable in the remaining patients. High-resolution HLA typing for all recipients and donors was performed as part of the clinical routine by NGS for HLA-A, B, C, DRB1, DRB3/4/5, DQA1, DQB1, and DPB1 (26). HLA antibody detection was also performed as part of the clinical routine using the Luminex Single Antigen bead assay for class I and class II (LABScreen Single Antigen, Thermo Fisher Scientific Inc., Waltham, MA). Detailed patient characteristics are provided in **Table 1**, and high-resolution HLA typings and DSA titers are available in **Supplementary Tables 1** and **2**, respectively. Recipient and donor peripheral mononuclear cells (PBMCs) were collected prior and within the first month as well as 3 and 12 months after transplantation and cryopreserved until analysis. The exact sampling timepoints for each patient are provided in **Supplementary Table 3**.

**Table 1:** Patients baseline characteristics.

Subject	Sex	Age at Tx	Cause of ESRD	MM	Type of Tx	DSA	Immunosuppressive therapy	CMV R/D
R154	Male	55	Unknown	2-1-1	DKD 2nd Tx	Class I; Cat 2	IAS + ATLG	-/-
R192	Female	60	IgA nephritis	1-1-0	DKD	Class I; Cat 3	IAS +ATLG	+/-
R200	Male	60	Diabetes type II	1-1-1	DKD	Class II; Cat 3	IAS + ATLG	+/-
R 294	Female	60	Unknown	1-1-0	DKD	Class II; Cat 2	IAS + ATLG	+/+
R 327	Male	59	ADPKD	1-1-2	DKD	Class II; Cat 2	IAS + ATLG	+/-
R 24 *^a^*	Male	54	ADPKD	1-1-0	LKD	No	IAS (7x) + PLEX(1x) + IL-2RA	+/-
R 30	Male	66	Amyloidosis	1-1-1	LKD	No	IL-2RA induction	+/+
R 190	Male	42	ADPKD	1-1-1	DKD	No	IL-2RA induction	-/-
R202	Female	65	ADPKD	0-1-1	DKD	No	IL-2RA induction	+/+
R211	Male	37	Unknown	1-1-0	DKD	No	IL-2RA induction	+/+

ADPKD, autosomal dominant polycystic kidney disease; IAS, immunoadsorption; MMF, mycophenolatmofetil; MM, HLA mismatch (A-B-DR); PLEX, plasma exchange; DKD, diseased kidney donation; LKD, living kidney donation.

Category 1: MFI 1000-2000; Category 2: MFI 2000-5000; Category 3: MFI 5000-10000.

a R24 had ABO incompatible transplantation.

Pre-sensitized recipients received an induction therapy with rabbit ATLG (Grafalon^®^) at a dose of 1.5 mg/kg for 7 days and concomitant immunoadsorption with GAM-146 peptide (Globaffin^®^) columns. Induction therapy for non-sensitized recipients was uniformly basiliximab (Simulect^®^). This reduced ATLG dosage for induction therapy in DSA-positive patients followed our center policy and is equally effective in prevention of acute cellular rejection when compared to higher ATLG dosing (27). Further immunosuppression was uniformly corticosteroids, mycophenolate-mofetil, and tacrolimus (TAC). TAC trough levels for all patients throughout the observation period are provided in **Supplementary Table 4**.

Mixed lymphocyte reaction (MLR) and RNA-based TCR sequencing were performed for all included patients. Additional phenotypical characterization of T-cells by FACS was performed for the group of patients receiving ATLG induction. The TCR repertoire was compared at the four defined timepoints between these two groups of induction therapy. Institutional ethics committee approval (EC NB: 267/2011) was obtained for all aspects of the study, and all study participants were included after signed informed consent prior to transplantation.

### Defining the T-Cell Repertoire

#### Phenotypic Characterization of T-Cells

Cryopreserved PBMCs were rapidly thawed and resuspended in pre-warmed RPMI 1640 (Gibco, Grand Island, NY, USA) containing 50 μg/ml DNASE I (Roche, Basel, Switzerland). Subsequently, cells were collected by centrifugation, resuspended in phosphate-buffered saline (PBS) (Gibco), and stained using DURAClone IM T Cell Subset tubes (Beckman Coulter). As DURAClone tubes are designed to be used with whole blood, additional preparatory steps were necessary for the use with isolated PBMCs. Dried reagents in the DURAClone tubes were reconstituted in 50 μl PBS and 5 μl of BV785-labeled anti-CD103 (BioLegend, San Diego, CA, USA), and 5 μl of BV605-labeled anti-CD31 (BioLegend) antibodies was added as additional antibodies. 30 μl of the resulting antibody mixture was used to stain 7 × 105 cells in a total volume of 50 μl. Staining was performed according to the manufacturer’s instruction skipping the red blood cell lysis step.

Cells were acquired on a Cytek Aurora (Cytek Biosciences, Fremont, CA, USA), and unmixed data were reanalyzed using Kaluza (Beckman Coulter, Brea, CA, USA). Numbers for the following T-cell phenotypes were recorded: CD4 T-cells (CD3+ CD4+), CD8 T-cells (CD3+ CD8+), CD4 recent thymic emigrants (CD3+, CD4+, CD31+), CD4- and CD8-naive T-cells (CD3+, CD4+/CD8+, CCR7+ CD45RA+), CD4 and CD8 central memory T-cells (CD3+, CD4+/CD8+, CCR7+ CD45RA-), CD4 and CD8 effector memory T-cells (CD3+, CD4+/CD8+, CCR7- CD45RA-), and CD4 and CD8 effector memory T-cells re-expressing CD45RA (CD3+, CD4+/CD8+, CCR7- CD45RA+). The gating strategy exemplified on one representative individual is provided in **Supplementary Figure 1**.

#### Mixed Lymphocyte Reaction

Mixed lymphocyte reactions were performed as previously described, reflecting T-cell activation driven mostly by the direct pathway of allorecognition (23, 28). Briefly, after thawing of cryopreserved PBMCs collected prior to transplantation, MLRs were performed by plating 2 × 10^5^ carboxyfluorescein succinimidyl ester (CFSE)-labeled (Invitrogen cat. #C34554) transplant recipient cells and 2 × 10^5^ violet proliferation dye (VPD)-labeled (BD Horizon cat. #562158) irradiated donor cells in each well of a 96-well plate. MLR cultures were incubated at 37°C for 6 days and were sorted on a FACS Aria Fusion high-speed cell sorter for CD4+ CFSE^low^ and CD8+ CFSE^low^ T-cells (BD Pharmingen, San Diego, CA, USA, cat.# 552852; BioLegend, San Diego, CA, USA, cat.# 317426; BD Pharmingen cat.# 557834). Unstimulated pre- and post-transplant samples were sorted for CD4+ and CD8+ T-cells to define the bulk repertoire. The sorting of T-cell populations was followed by RNA isolation and library preparation for TCR sequencing. A schematic overview of the method for identifying donor-reactive TCRs are found in **Supplementary Figure 2**.

#### TCR Repertoire Sequencing

RNA isolation, NGS library preparation, sequencing, and bioinformatics analysis were previously described (29). In brief, lymphocytes were sorted from PBMCs, and RNA isolation was done following the original TRIzol protocol (Invitrogen, Carlsbad, CA). TCR libraries were sequenced on an Illumina NextSeq 500. Barcodes and unique molecular identifiers were processed with MIGEC (21), and for clonotype assembly MiXCR (30) was employed.

#### Virus-Specific TCRs

Virus-specific TCRs were identified by matching the CDR3 sequence against TCRs with known specificity available in the VDJdb database using VDJmatch (31) (https://github.com/antigenomics/vdjmatch).

### Statistical Methods

The statistical methods used to examine the repertoires with regard to donor-reactive clonotype distribution, diversity, and similarity between repertoires followed the procedure previously described in depth in (23).

#### Repertoire Processing

Each repertoire comprised the following information per clonotype: the CDR3 nucleotide and amino acid sequence, reads (clones), frequency, and the V, D, and J genes. Ambiguity correction for CD4 and CD8 T-cells was carried out by assigning ambiguous clonotypes to the phenotype with twice the number of clones. Remaining ambiguous clonotypes were removed from the repertoire, and corresponding frequency adjustments were carried out. Clonotypes were defined as donor-reactive in case of a fold change equal or greater than five with regard to the reads between the unstimulated and the stimulated pre-transplant sample. This fold expansion criterion has been used in previous studies also employing MLRs for defining the donor-specific repertoire to exclude bystander activation that may occur during the later phase of the MLR (22, 23).

Further, all T-cell repertoires were downsampled to the lowest read count within each phenotype group to avoid immediate effects of sequencing depth on the findings of the statistical analyses. Hence, all presented results are based on the mean estimates obtained from 1,000 downsampled repertoires.

#### Diversity and Statistical Analysis

Diversity of each repertoire was measured in terms of (i) R20, the fraction of clonotypes (ordered by decreasing frequency) which constitute 20% of the repertoire, and (ii) clonality, defined as 1-P where P denotes Pielous’s index which is the ratio between the observed and the maximum possible Shannon’s entropy (20). Continuous variables are presented as mean ± standard deviation (SD). Differences between the ATLG and the basiliximab group were evaluated with unpaired t-tests. The paired t-test was used to determine differences in findings between different follow-up timepoints. Mixed factorial ANOVA was used for the simultaneous assessment of differences of the between-subject factor treatment and the within-subject factors of time with regard to a continuous variable (e.g., diversity). Differences were considered significant at a p-value of less than 0.05. p-values were not adjusted for multiple testing due to the exploratory nature of the statistical analysis. Statistical analysis and comparisons were performed with the statistical software R version 4.0.2 (R Foundation for Statistical Computing, Vienna, Austria).

#### Repertoire Overlap

Similarity analysis to compare the overlap between the T cell repertoires in terms of CDR3 nucleotide sequences, VJ pairing, and V and J usage included (i) the Jensen–Shannon divergence (JSD), a similarity measure quantifying the divergence between two probability distributions, and (ii) graphical assessment by circos plots visualizing the differences in frequencies of VJ parings (32).

## Results

### Overall and Donor-Reactive TCR Repertoire

Following induction therapy after kidney transplantation, a lymphodepletion in the ATLG group reaching a mean of 0.11 + -0.07 G/L over the first month was observed. The lymphopenic state in our cohort lasted for at least 14 days, and lymphocyte numbers recovered to >1 G/L in two patients at day 310 and three subjects presented with prolonged reduced lymphocyte counts throughout the observation period. Lymphocyte counts in the basiliximab group also decreased in the first month after transplantation but rose to >1 G/L in all subjects in subsequent timepoints (**Table 2**). Graft function determined by estimated glomerular filtration rate (eGFR) at 12 months after transplantation was not different between the groups (ATLG group: 64 +- 18 ml/min/1.73 m², basiliximab group: 53 +- 12 ml/min/1.73 m²; p = 0.23).

**Table 2:** Time-specific distribution of lymphocyte numbers.

Treatment group	PreTX	1M	3M	12M	Ref
ATLG	1.21 + -0.28	0.11 + -0.07	0.81 + -0.43	0.87 + -0.43	1–4 G/L
Basiliximab	1.27 + -0.38	0.74 + -0.51	1.87 + -0.85	1.90 + -1.04	1–4 G/L
P	0.795	0.051	0.048	0.093	

ATLG caused a lymphodepletion in the first months after transplantation.

**Figure 1:**
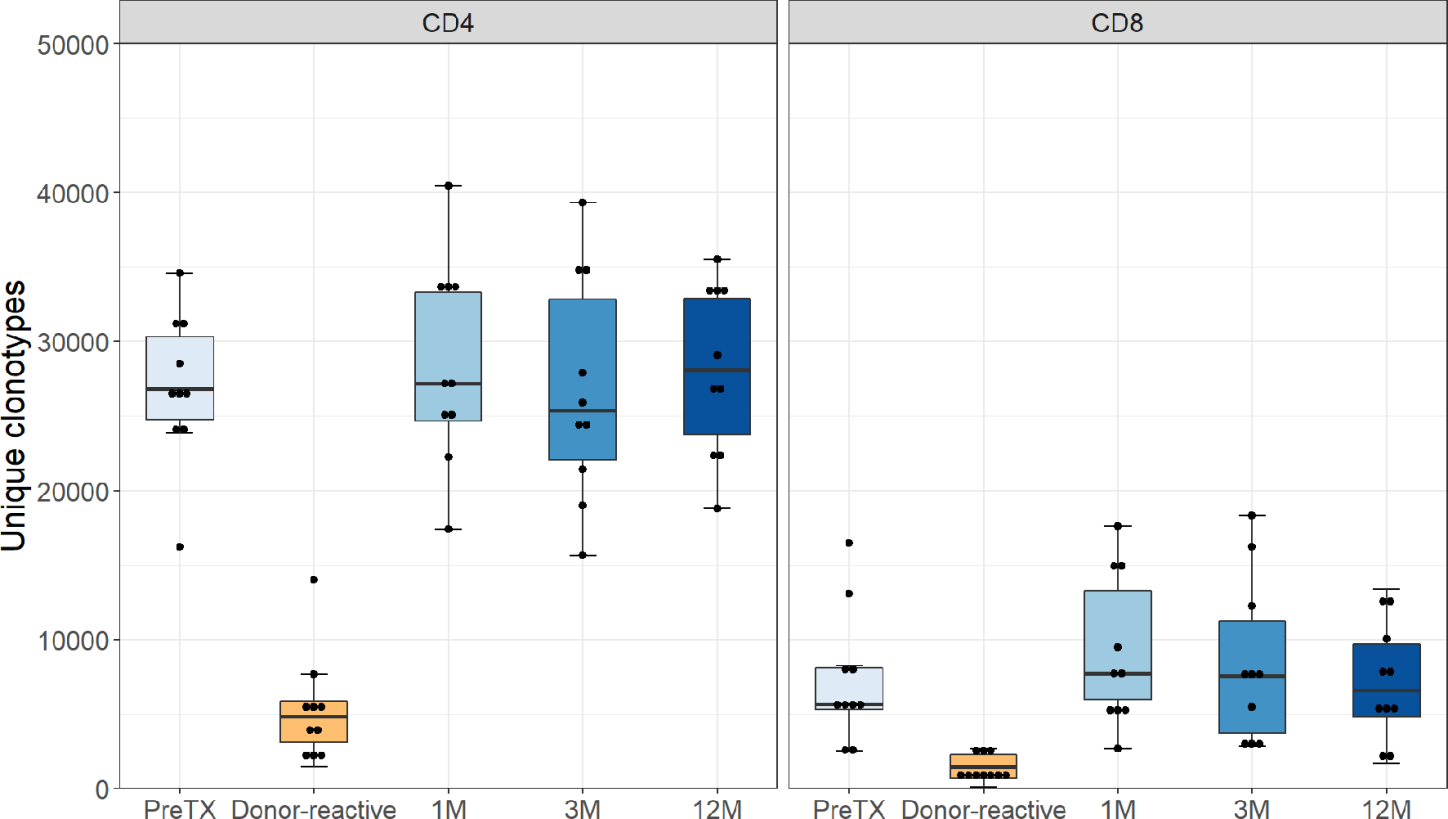
Number of unique clonotypes after normalization by downsampling to the smallest number of reads. Grouped by phenotype groups CD4 and CD8 for the pre-transplant, the donor-reactive repertoire, and samples from the consecutive timepoints. The number of detected TCR clonotypes was comparable to pre-transplant in both groups at all timepoints.

The absolute number of detected TCR clonotypes after transplantation *via* NGS sequencing of the TCR beta chain was comparable to pre-transplant in both groups at all timepoints and ranged from 26,328 to 107,735 for CD4 and 7,184 to 87,533 for CD8 T-cells. The numbers of clonotypes remaining after downsampling are shown in **Figure 1**. The donor-reactive repertoire defined following MLR revealed a lower number of clonotypes than the unstimulated repertoires in both cohorts, as expected. Detailed numbers of clonotypes and clones are reported in the **Supplementary Tables 5** and **6**.

Phenotypic characterization of T-cells by FACS analysis in the group of pre-sensitized patients receiving ATLG treatment revealed a high heterogeneity of the patients in terms of their immune cell composition. Patients had decreased numbers of naive T-cells and elevated numbers of central memory CD4 T-cells prior to transplantation. The majority of patients showed a decline in recent thymic emigrants, indicating that reduced-dose ATLG was sufficient to achieve lymphodepletion in the thymus. An analysis of CD4 and CD8 T-cells revealed features consistent with lymphodepletional induction therapies, yet inversion of the CD4/CD8 ratio was, when observed at all, only minimal (**Supplementary Figures 3-6**).

### TCR Repertoire Characteristics

**Figure 2:**
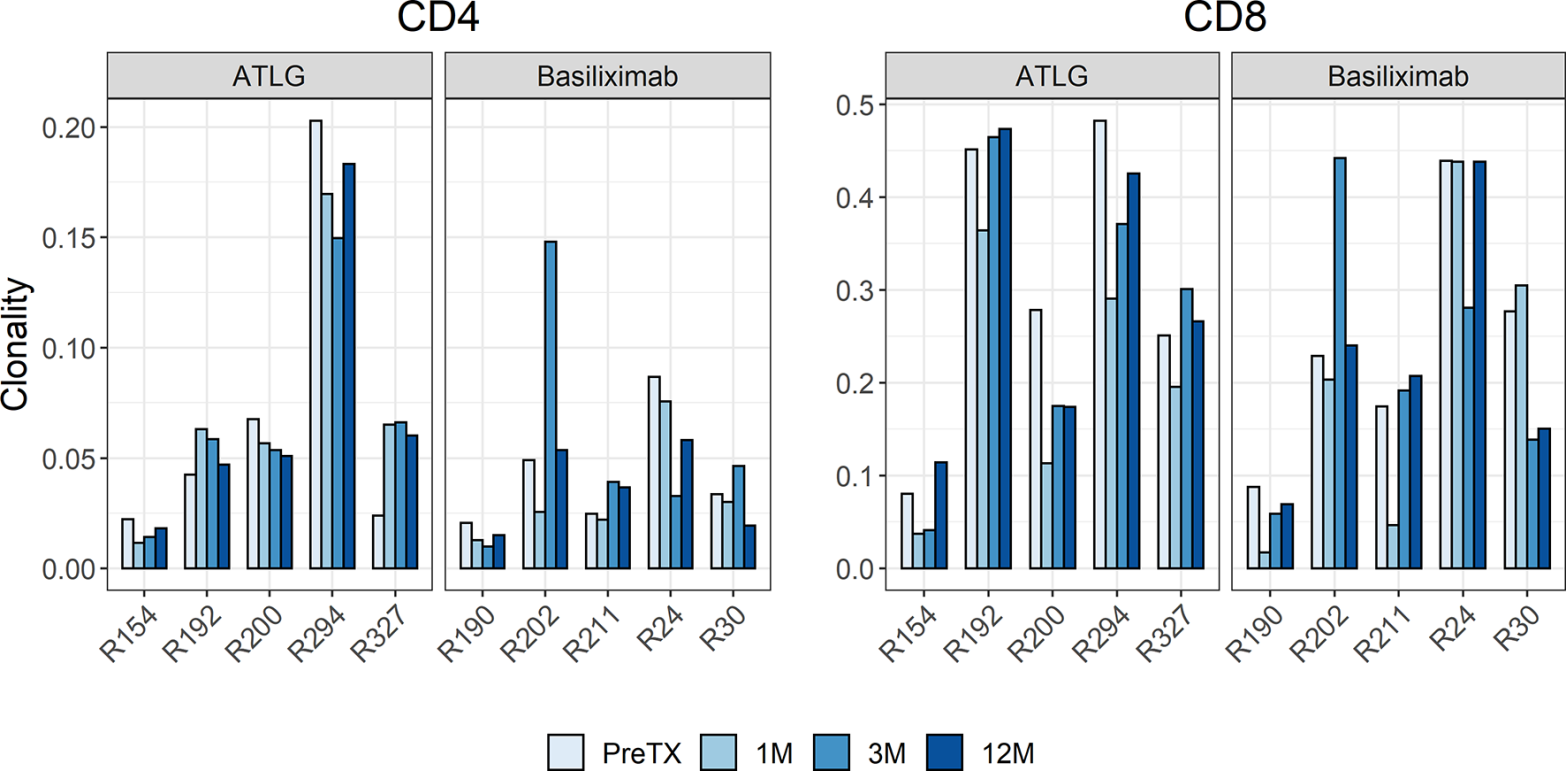
Barchart of clonality estimates of the pre- and post-transplant repertoires grouped by CD4 and CD8 cells. The clonality of post-transplant samples showed no statistical difference between the ATLG and the basiliximab group.

The diversity of the TCR repertoires showed a wide individual variability. Between the two groups, there was no difference in clonality pre-transplant with a mean of 0.07 for CD4 and 0.31 for CD8 T-cells in the ATLG group and a mean of 0.04 for CD4 and 0.24 for CD8 T-cells in the basiliximab group. The clonality of post-transplant samples kept the individual variability and showed no statistical difference between the two groups over time in both CD4- and CD8-positive T-cells, respectively (CD4: F(1.45, 11.6) = 0.64 p = 0.496; CD8: F(3, 24) = 0.60 p = 0.620) (**Figure 2**).

**Figure 3:**
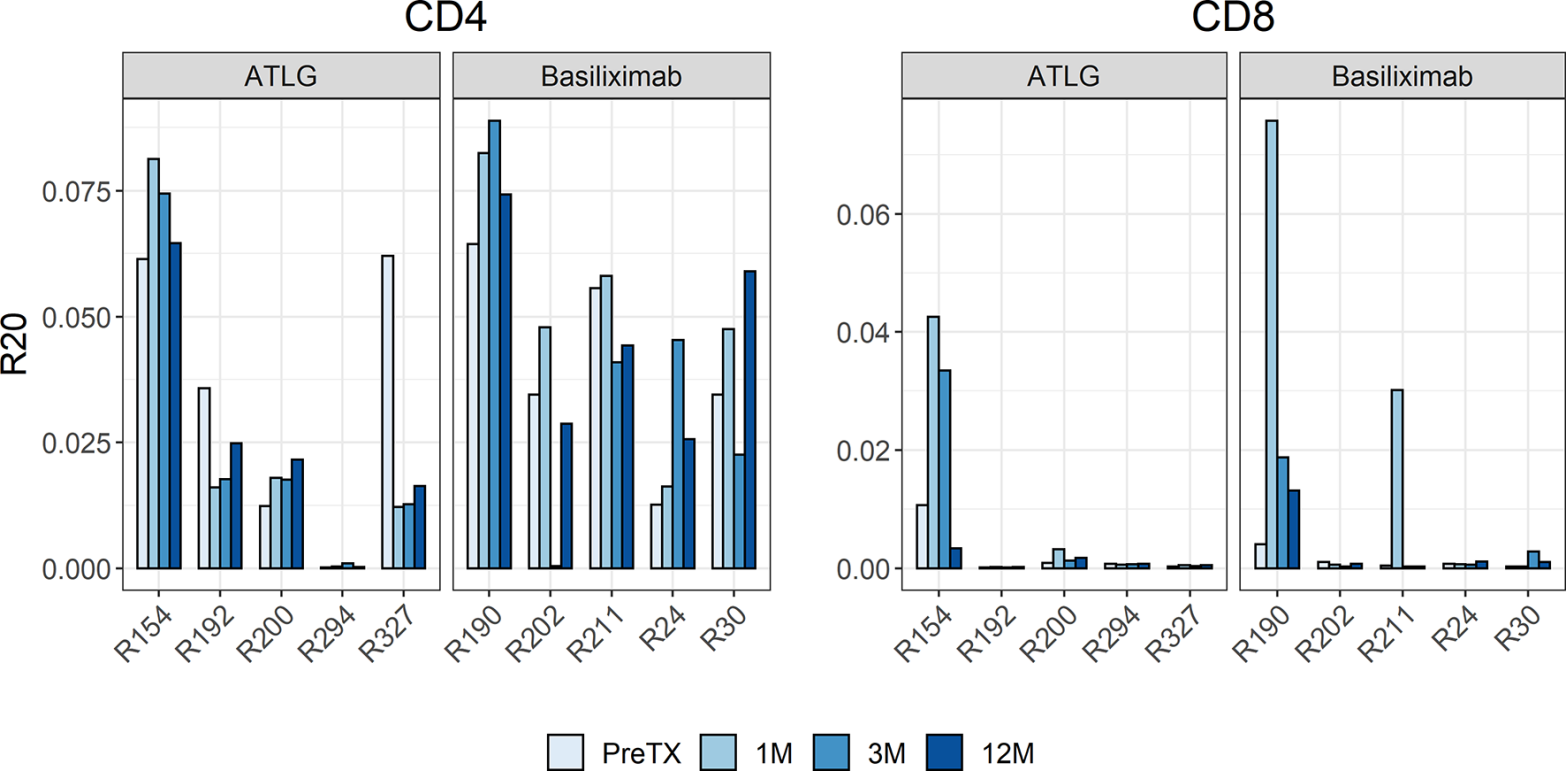
Barchart of R20 estimates of the clonotypes in the pre- and post-transplant repertoires for CD4 and CD8 cells. The individual repertoire changes indicated no increased clonal dominance in the overall TCR repertoire following reduced-dose ATLG treatment.

The R20 measure, a metric for immune dominance, showed no segregation of the two cohorts indicating no increased clonal dominance in the overall TCR repertoire following reduced-dose ATLG treatment (**Figure 3**). The R20 of CD4- and CD8-positive T-cells after induction therapy was stable, and no statistical difference was observed in all timepoints after transplantation (CD4: F(3, 24) = 0.85 p = 0.479; CD8: F(1.19, 9.52) = 0.79 p = 0.418). A highly abundant clone in one subject in the ATLG-treated group (R294) represented more than 10% of the entire T-cell repertoire and was found in every timepoint pre- and post-transplant, responsible for the low R20 values in this patient.

**Figure 4:**
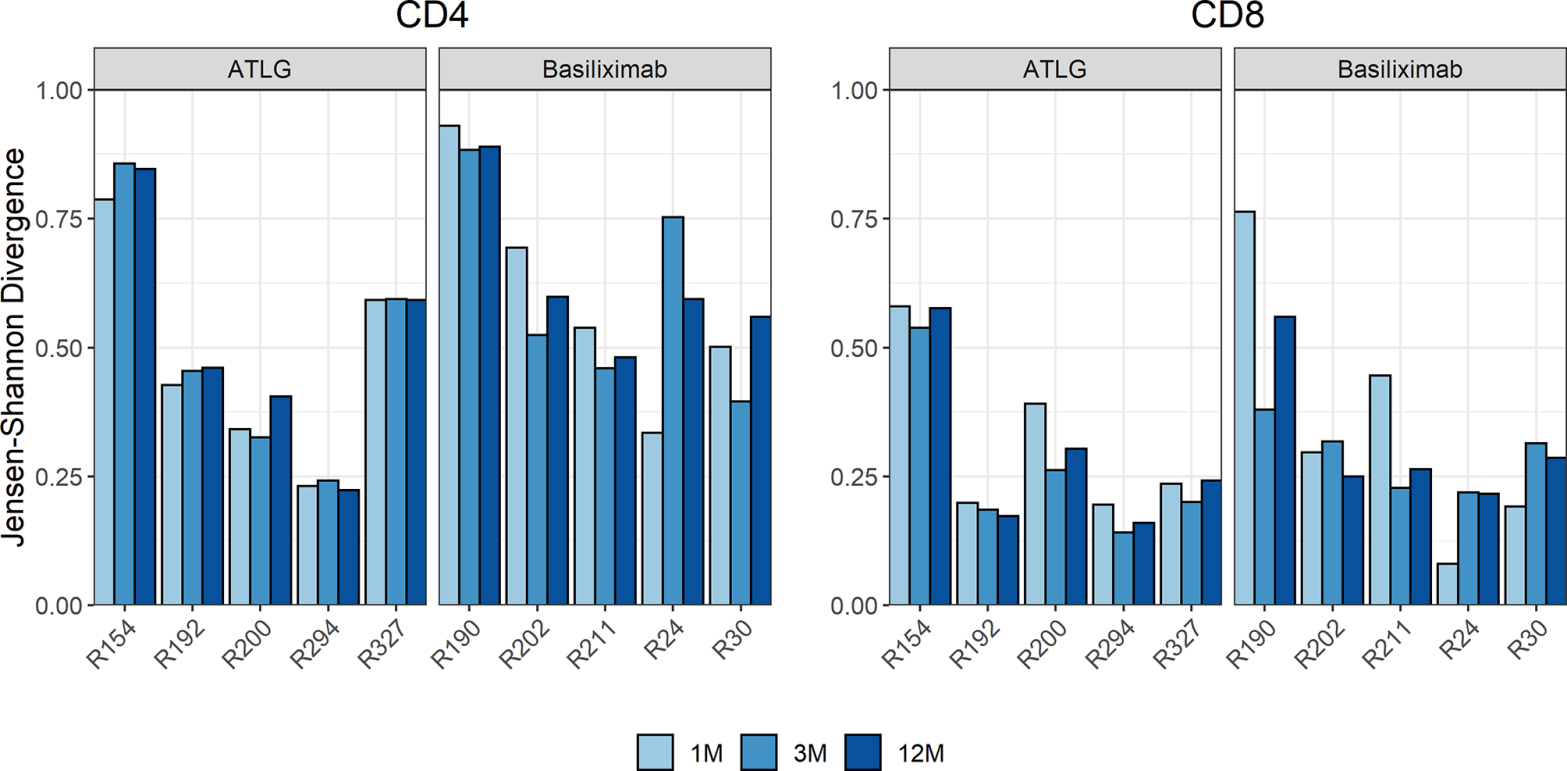
Time course of the Jensen–Shannon divergence (JSD) of the top 1,000 clones compared to baseline. No statistical difference was observed after transplantation, indicating that ATLG patients did not experience a higher repertoire turnover than basiliximab patients after transplantation.

We quantified if overall turnover in the TCR repertoire differed during lymphocyte reconstitution in the ATLG group compared to patients in the basiliximab group, which would be indicated by a high JSD. The measured JSD values were yet similar in the two groups at all timepoints post-transplant (**Figure 4**). This indicates that ATLG patients did not experience a higher repertoire turnover than basiliximab patients after transplantation.

**Figure 5:**
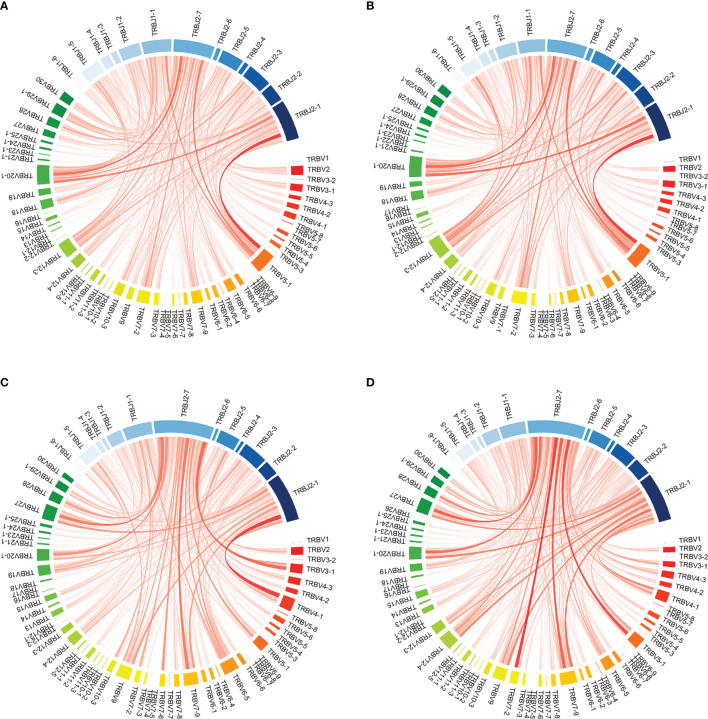
Visualization of VJ combination usage in patient R154 from the reduced-dose ATLG induction group prior to transplantation and in the first month post transplantation. **(A, D)** show VJ combination usage in the full CD4 and CD8 TCR repertoires at the two timepoints: **(A)** PreTX CD4; **(B)** 1M CD4; **(C)** PreTX CD8; **(D)** 1M CD8.

Likewise, the change in VJ combination usage in the TCR repertoire pre- and post-induction treatment was similar between the two groups. Compared to the overall TCR repertoire, turnover divergence in VJ combination from baseline was low with a mean JSD of 0.02 and 0.07 for CD4 and CD8, respectively. VJ combination usage prior to transplantation and in the first month post-transplant from one representative patient in the ATLG induction group is shown in **Figure 5**. Quantification of TCRs with known specificities for Cytomegalovirus (CMV) or Epstein–Barr virus (EBV) both being responsible for common post-transplant viral infections showed that the number of virus-specific T-cells was comparable between the two treatment groups and remained stable over time (CMV: CD4: F(3, 24) = 0.76 p = 0.530; CD8: F(3, 24) = 0.27 p = 0.849; EBV: CD4: F(3, 24) = 1.64 p = 0.206; CD8: F(1.13, 9.03) = 1.32 p = 0.287). Compared to the pre-transplant T-cell repertoires, donor-reactive CD4 T-cell repertoires showed an increased abundance of CMV-specific T-cells (t(9) = -2.9 p = 0.017). Cumulative frequencies of virus specific T-cells are provided in **Supplementary Figure 7**.

### The Donor-Reactive T-Cell Repertoire

**Figure 6:**
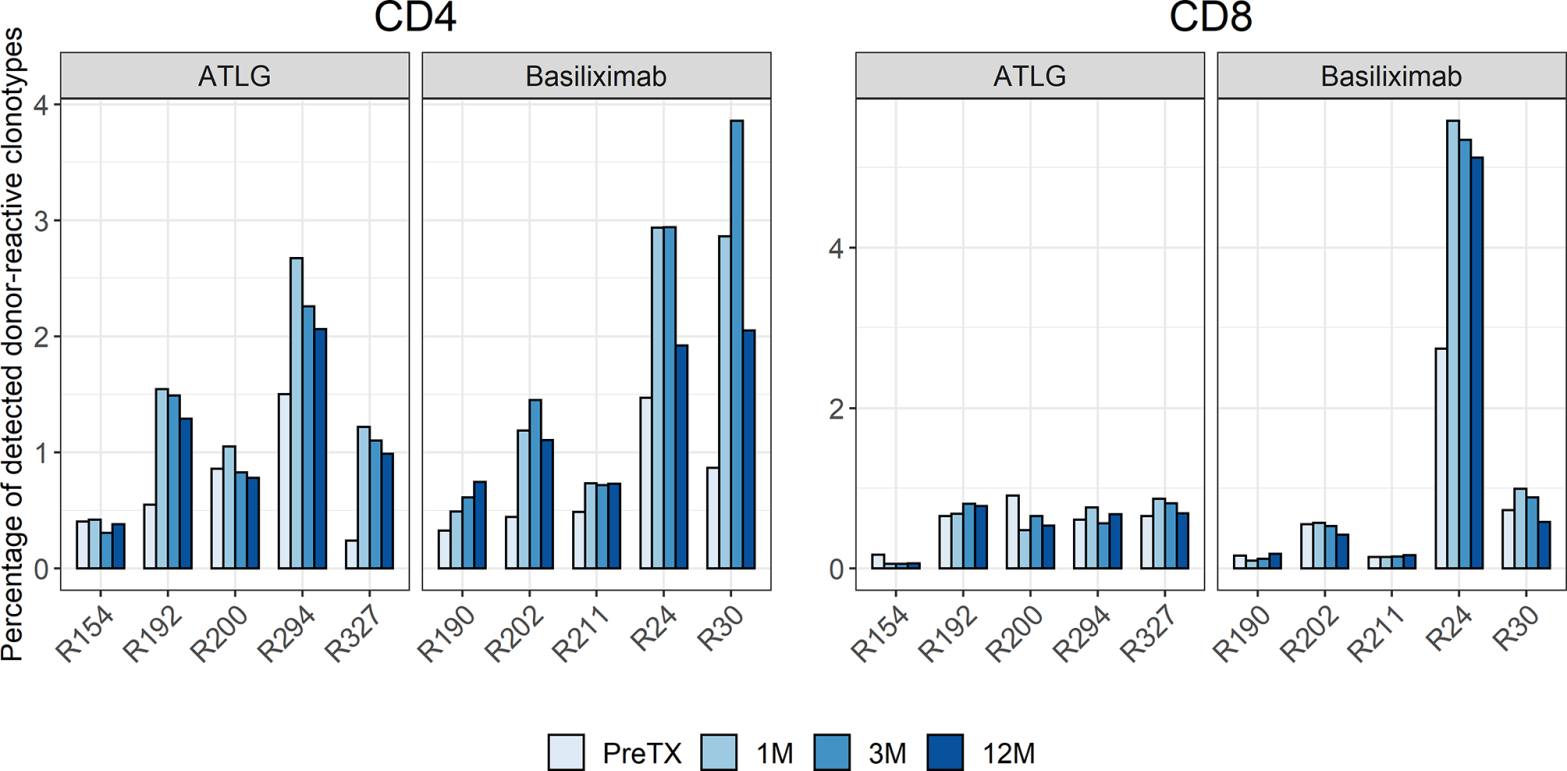
Percentage of detected donor-reactive clonotypes after normalization by downsampling to the smallest number of reads. An increase in donor-reactive clonotypes early after transplantation was seen in the CD4 T-cell repertoire of the basiliximab group and the majority of patients in the ATLG group.

The preformed donor-reactive TCR repertoire was defined pre-transplant. Analysis of preformed donor-reactive clonotypes detectable among all clonotypes in the bulk T-cell repertoires pre- and post-transplant revealed a significant increase in the percentage of detectable CD4 donor-reactive clonotypes after transplantation irrespective of the induction therapy (t(9) = -3.89 p = 0.004). In the ATLG group, the percentage of CD4 donor-reactive clonotypes increased on average by 0.67% to a total of 1.38% ± 0.83 after transplantation. Likewise, in the basiliximab group an average increase of 0.92% to a total of 1.64% ± 1.17 of CD4 donor-reactive clonotypes was observed after transplantation. The percentage of donor-reactive clonotypes in the bulk repertoires of all patients at all timepoints is visualized in **Figure 6** and are shown in **Supplementary Table 7**. A numerical increase in donor-reactive clonotypes was seen in CD4 T-cells in all subjects in the basiliximab group over the observed time post-transplant, as expected. In the ATLG group, however, a less marked increase of donor-reactive cells in two individuals was of interest potentially caused by a prolonged lymphopenia in these subjects.

Contrary to the CD4 donor-reactive clonotypes, no statistically significant change in the percentage of CD8 donor-reactive clonotypes was observed after transplantation (t(9) = -1.01 p = 0.341).

Overall, the percentage of donor-reactive clonotypes was similar in both treatment groups throughout the post-transplant observation period (CD4: F(1.26, 10.08) = 1.38 p = 0.279; CD8: F(1.06, 8.48) = 1.15 p = 0.319).

This increase of the donor-reactive repertoire was not only observed on a clonotype level but also on the level of frequencies of circulating donor-reactive T-cells after transplantation. For CD4 donor-reactive T-cells, a significant increase after transplantation was observed (t(9) = -3.59 p = 0.006), whereas the percentage of CD8 donor-reactive T-cells remained similar after transplantation (t(9) = 1.57 p = 0.150). The mean increase of CD4 donor-reactive T-cells after transplantation in the ATLG and basiliximab group was 0.90% and 0.89%, respectively.

## Discussion

In this study, we showed that reduced-dose ATLG induction therapy in sensitized kidney transplant recipients led to sufficient lymphodepletion without excessive clonal expansion of alloreactive T-cells. Excessive shifts in the overall diversity of the repopulating TCR repertoire were not seen and comparable to patients receiving basiliximab induction. Properties of the TCR repertoires, such as clonality and R20, exhibited between patient variability. This was, however, not unexpected as also a previous study performed in healthy individuals observed interindividual differences in clonality and diversity that persisted over time (33). Such differences may reflect an individual’s history of antigen exposure.

We detected an appearance of the donor-reactive T-cells already during the lymphopenic state as soon as 10 days after transplantation. Donor-reactive T-cells at similar rates were also present in patients receiving basiliximab, yet these patients present with normal lymphocyte counts after transplantation. The increase in donor-reactive T-cells in both groups did not affect the diversity of the overall TCR repertoire or repertoire turnover during or after lymphopenia resolved in the ATLG-treated subjects. This demonstrates that reduced-dose ATLG in pre-sensitized individuals leads to similar TCR repertoire changes as in non-sensitized basiliximab-treated patients with no inflation of CMV- or EBV-specific T-cells.

However, we observed an overrepresentation of CMV-specific T-cells in the donor-reactive T-cell repertoires. The existence of shared TCR sequences between CMV and donor-reactive T-cells in HLA class I mismatched kidney transplant recipients has been described before (34).

At the phenotypic level, previously described changes after lymphodepletion were only observed to a lesser extent in this study. An expected skewed TCR repertoire and repopulation of the peripheral lymphocyte repertoire by a restricted set of lymphocytes following lymphopenia was not observed after reduced-dose ATLG.

Previous studies described an inversion of the CD4/CD8 ratio, a more pronounced decrease of effector and naïve T-cells compared with cells of a memory phenotype, and a prolonged T-cell dysfunction and reported a more severe and sustained lymphopenia (5, 11–18).

This is most likely due to higher doses, ranging from 4 mg/kg per day to up to 9 mg/kg at transplantation followed by 3 mg/kg for 4 days, of the lymphodepleting antibody, and the majority of studies have been conducted with ATG (Thymoglobuline^®^), an anti-thymocyte globulin with different properties than ATLG. Thymoglobuline is produced by immunizing rabbits with fresh human thymocytes, and ATLG on the other hand is obtained by immunization of rabbits with Jurkat T-cells suggesting a narrower spectrum of targeted antigens by ATLG compared to Thymoglobuline^®^ (9). Comparable studies using reduced dosages of lymphodepletional antibodies were almost uniquely performed with Thymoglobuline, but likewise a lesser degree of T-cell depletion was observed with smaller doses (6, 35–37).

A limitation of this study is a small sample size; however, the detailed follow-up over 1 year is certainly sufficient to cover the range of immune reconstitution after lymphodepletion (no statistically significant difference in lymphocyte counts at the end of the observation period was observed between the groups), and all ATLG-treated patients were pre-sensitized and received identical maintenance immunosuppression.

The strength of this paper is the detailed characterization of lymphocyte repopulation following lymphodepletional induction therapy by TCR sequencing including not only the entire circulating TCR repertoire but also the donor-specific T-cells of each patient.

Based on the presented data, we conclude that the repopulated TCR repertoire in kidney transplant recipients after reduced-dose ATLG is not narrowed or skewed leading to comparable overall diversity and T-cell turnover as in patients receiving basiliximab. Likewise, donor-reactive T-cells increase after a reduced-dose ATLG treatment in a similar way to basiliximab induced kidney transplant recipients.

Reduced-dose ATLG preserves the shape of the TCR repertoire, potentially leading to a lower susceptibility for infections or cancer without an increase of rejection episodes.

## Data Availability Statement

The datasets presented in this study can be found in online repositories. The names of the repository/repositories and accession number(s) can be found as follows: https://ega-archive.org, EGAD00001008478.

## Ethics Statement

The study was reviewed and approved by the ethics committee of the Medical University of Vienna (EC NB: 267/2011). The patients provided their written informed consent to participate in this study.

## Author Contributions

RO, AH, and CA were responsible for the conception, design, financial support, critical revision, and final approval of the manuscript. RO, CA, KJ, and AH were responsible for the manuscript writing. KJ performed the library preparation for subsequent sequencing of all samples. KJ and MG prepared the figures. MG and AH were responsible for the statistics, bioinformatic analysis, and revision of the manuscript. KH, AH, and CA performed the sample collection, *in vitro* experiments, FACS sorting, and data analysis. RR-S, KH, SW, and TW were responsible for the critical revision of the manuscript. All authors contributed to the article and approved the submitted version.

## Funding

The study was founded by the Scientific Funds of the Austrian National Bank [OeNb project number 17289 (https://www.oenb.at)], the WWTF (Vienna Science and Technology Fund, grant# LS20-081), and the Medical University of Vienna Transplantation Research Platform’s Start-Up Grant 2020. The funding bodies had no influence on the design, collection, analysis, and interpretation of data and writing the manuscript.

## Conflict of Interest

The authors declare that the research was conducted in the absence of any commercial or financial relationships that could be construed as a potential conflict of interest.

## Publisher’s Note

All claims expressed in this article are solely those of the authors and do not necessarily represent those of their affiliated organizations, or those of the publisher, the editors and the reviewers. Any product that may be evaluated in this article, or claim that may be made by its manufacturer, is not guaranteed or endorsed by the publisher.

## Abbreviations

ANOVA, analysis of variance; ATG, anti-thymocyte globulin; ATLG, anti-T-lymphocyte globulin; CDR3, complementarity determining region 3; CFSE, carboxyfluorescein succinimidyl ester; CMV, cytomegalovirus; DSA, donor-specific anti-HLA antibody; EBV, Epstein–Barr virus; ELISPOT, enzyme-linked immunospot; FACS, fluorescence-activated cell sorting; MFI, mean fluorescence intensity; MIGEC, Molecular Identifier Guided Error Correction pipeline; MLR, mixed lymphocyte reaction; NK cells, natural killer cells; PBS, phosphate-buffered saline; TAC, tacrolimus; TCR, T-cell receptor; UMI, unique molecular identifier; V(D)J, Variable, Diversity, Joining; VPD, Violet proliferation dye.
