# Supplementary material for: Effects of Reduced-Dose Anti-Human T-Lymphocyte Globulin on Overall and Donor-Specific T-Cell Repertoire Reconstitution in Sensitized Kidney Transplant Recipients
Source: Front Immunol. 2022 Feb 25;13:843452. doi: 10.3389/fimmu.2022.843452 (PMC8913717; doi:10.3389/fimmu.2022.843452)
Supplement: Supplementary file 1 [file DataSheet_1.docx]

Supplementary Material

| **Patient** | **recipient/donor** | **HLA-A*** | **HLA-A*** | **HLA-B*** | **HLA-B*** | **HLA-C*** | **HLA-C*** | **HLA-DRB1*** | **HLA-DRB1*** | **HLA-DRB** | **HLA-DRB** | **HLA-DQB1*** | **HLA-DQB1*** | **HLA-DQA1*** | **HLA-DQA1*** | **HLA-DPB1*** | **HLA-DPB1*** |
| --- | --- | --- | --- | --- | --- | --- | --- | --- | --- | --- | --- | --- | --- | --- | --- | --- | --- |
| **R154** | rec | 30:01 | 31:01 | 07:02 | 51:01 | 07:02 | 15:02 | 04:01 | 04:07 | 4*01:03 |  | 03:01 | 03:02 | 03:03 |  | 03:01 | 04:01 |
|  | do | 02:01 | 03:01 | 07:02 | 15:01 | 03:03 | 07:02 | 04:01 | 11:03 | 3*02:02 | 4*01:03 | 03:01 | 03:02 | 03:01 | 05:05 | 03:01 | 04:02 |
| **R192** | rec | 23:01 | 33:03 | 15:17 | 49:01 | 07:01 |  | 11:01 | 13:02 | 3*02:02 | 3*03:01 | 03:01 | 06:04 | 01:02 | 05:05 | 04:01 |  |
|  | do | 01:01 | 30:01 | 13:02 | 15:17 | 06:02 | 07:01 | 13:01 | 13:03 | 3*01:01 | 3*02:02 | 03:01 | 06:03 | 01:03 | 05:05 | 03:01 | 04:01 |
| **R200** | rec | 11:01 | 33:03 | 13:01 | 35:03 | 04:01 | 04:03 | 11:01 | 15:01 | 3*02:02 | 5*01:01 | 05:02 | 06:01 | 01:02 |  | 04:02 | 14:01 |
|  | do | 11:01 | 24:02 | 07:02 | 35:01 | 04:01 | 07:02 | 04:02 | 15:01 | 4*01:03 | 5*01:01 | 03:02 | 06:02 | 01:02 | 03:01 | 04:01 |  |
| **R294** | rec | 02:01 | 24:02 | 51:01 | 55:01 | 01:02 | 15:02 | 11:01 | 13:01 | 3*02:02 |  | 03:01 | 06:03 | 01:03 | 05:05 | 04:01 | 04:02 |
|  | do | 24:02 | 32:01 | 15:24 | 51:01 | 03:03 | 14:02 | 11:04 | 13:01 | 3*02:02 |  | 03:01 | 06:03 | 01:03 | 05:05 | 02:01 | 04:01 |
| **R327** | rec | 11:01 | 24:02 | 15:01 | 35:03 | 04:01 | 12:03 | 04:03 | 09:01 | 4*01:03 |  | 03:02 | 03:03 | 03:01 | 03:02 | 03:01 | 05:01 |
|  | do | 11:01 | 68:01 | 08:01 | 15:17 | 04:01 | 07:01 | 03:01 | 13:02 | 3*01:01 | 3*03:01 | 02:01 | 06:04 | 01:02 | 05:01 | 01:01 | 04:01 |
| **R24** | rec | 02:01 | 02:05 | 07:02 | 50:01 | 06:02 | 07:02 | 07:01 | 15:01 | 4*01:03 | 5*01:01 | 02:02 | 06:02 | 01:02 | 02:01 | 04:01 |  |
|  | do | 02:01 | 26:01 | 07:02 | 40:02 | 02:02 | 07:02 | 15:01 |  | 5*01:01 |  | 06:02 |  | 01:02 |  | 04:01 | 04:02 |
| **R30** | rec | 11:01 | 29:02 | 07:02 | 35:01 | 07:02 | 16:01 | 13:01 | 15:01 | 3*02:02 | 5*01:01 | 06:02 | 06:03 | 01:02 | 01:03 | 03:01 | 04:01 |
|  | do | 02:01 | 29:02 | 44:02 | 35:01 | 05:01 | 16:01 | 04:01 | 13:01 | 3*02:02 | 4*01:03 | 03:01 | 06:03 | 01:03 | 03:03 | 03:01 |  |
| **R190** | rec | 01:01 | 03:01 | 07:02 | 50:01 | 06:02 | 07:02 | 07:01 | 14:54 | 3*02:02 | 4*01:03 | 02:02 | 05:03 | 01:04 | 02:01 | 04:01 | 10:01 |
|  | do | 03:01 | 30:01 | 07:02 | 13:02 | 06:02 | 07:02 | 07:01 | 15:01 | 4*01:03 | 5*01:01 | 02:02 | 06:02 | 01:02 | 02:01 | 03:01 | 04:01 |
| **R202** | rec | 02:01 | 25:01 | 07:02 | 18:01 | 07:02 | 12:03 | 04:01 | 15:01 | 4*01:03 | 5*01:01 | 03:02 | 06:02 | 01:02 | 03:01 | 04:01 |  |
|  | do | 02:01 | 25:01 | 18:01 | 44:02 | 05:01 | 12:03 | 13:01 | 15:01 | 3*01:01 | 5*01:01 | 06:02 | 06:03 | 01:02 | 01:03 | 02:01 |  |
| **R211** | rec | 02:01 | 24:02 | 13:02 | 35:02 | 04:01 | 06:02 | 07:01 | 11:04 | 3*02:02 | 4*01:03 | 02:02 | 03:01 | 02:01 | 05:05 | 02:01 | 04:01 |
|  | do | 02:01 | 29:02 | 07:02 | 13:02 | 06:02 | 07:02 | 07:01 | 11:04 | 3*02:02 | 4*01:03 | 02:02 | 03:01 | 02:01 | 05:05 | 02:01 | 04:01 |

**Supplementary Table 1** High resolution HLA-typings of recipients and their respective donors. HLA mismatches according to the Eurotransplant HLA matching algorithm are highlighted in yellow.

|  |  |  | **First Occurrence** | | | **Closest Observation to 12 Months** | |
| --- | --- | --- | --- | --- | --- | --- | --- |
| **Patient** | **Group** | **Locus** | **Time** | **MFI** | **dnDSA** | **Time** | **MFI** |
| R154 | ATLG | A*02:01 | 3 | 1055 | n | 11 | - |
| R192 | ATLG | A*01:01 | 0 | 17685 | n | 15 | 1893 |
| R192 | ATLG | B*13:02 | 0 | 4435 | y | 15 | - |
| R200 | ATLG | DPB1*04:01 | 12 | 1612 | y | 12 | 1612 |
| R294 | ATLG | - | - | - | - | 13 | - |
| R327 | ATLG | DRB3*03:01 | 0 | 1942 | n | 11 | 2186 |
| R327 | ATLG | DQB1*02:01 | 11 | 1334 | y | 11 | 1334 |
| R24 | Basiliximab | - | - | - | - | 13 | - |
| R30 | Basiliximab | DRB4*01:03 | 1 | 1867 | y | 6 | 1594 |
| R190 | Basiliximab | - | - | - | - | 10 | - |
| R202 | Basiliximab | - | - | - | - | 15 | - |
| R211 | Basiliximab | - | - | - | - | 15 | - |

**Supplementary Table 2** DSA follow up for patients in both treatment groups throughout the first-year post-transplant. Provided is the time point of first DSA occurrence together with the mean fluorescence intensity (MFI) and classification into preformed or de-novo DSA (dnDSA) as well as the MFI at the observation closest to one-year post-transplant. Dashes indicate that no DSA with MFI > 1,000 was detectable. To improve readability rows for every other patient have a grey background.

| **Patient** | **PreTX** | **1M** | **3M** | **12M** |
| --- | --- | --- | --- | --- |
| R24 | 0 | 6 | 83 | 356 |
| R30 | 0 | 41 | 107 | 356 |
| R154 | 0 | 29 | 88 | 513 |
| R190 | 0 | 7 | 81 | 517 |
| R192 | 0 | 31 | 82 | 463 |
| R200 | 0 | 27 | 99 | 419 |
| R202 | 0 | 26 | 93 | 320 |
| R211 | 0 | 9 | 106 | 478 |
| R254 | 0 | 15 | 97 | 385 |
| R294 | 0 | 10 | 108 | 356 |
| R327 | 0 | 36 | 122 | 275 |

**Supplementary Table 3** Exact timepoints of sample acquisition. For each patient the days post-transplant on which the samples were collected are provided.

| **Sample** | **Group** | **1M** | **3M** | **12M** |
| --- | --- | --- | --- | --- |
| R154 | ATLG | 11.2 | 7.6 | 6.6 |
| R192 | ATLG | 12.3 | 12.4 | 9.4 |
| R200 | ATLG | 13.0 | 10.1 | 7.5 |
| R294 | ATLG | 12.3 | 6.6 | 6.7 |
| R327 | ATLG | 12.4 | 14.4 | 6.5 |
| R24 | Basiliximab | 10.5 | 9.0 | 6.1 |
| R30 | Basiliximab | 8.9 | 9.5 | 6.6 |
| R190 | Basiliximab | 8.2 | 6.2 | 6.2 |
| R202 | Basiliximab | 10.8 | 6.3 | 7.1 |
| R211 | Basiliximab | 8.6 | 8.4 | 7.3 |

**Supplementary Table 4** TAC trough levels throughout the observation period.

| **Patient** | **Timepoint** | **Group** | **Clones - CD4** | **Clonotypes - CD4** | **Clones - CD8** | **Clonotypes - CD8** |
| --- | --- | --- | --- | --- | --- | --- |
| R154 | PreTX | ATLG | 141962 | 49620 | 48812 | 10540 |
| R154 | Donor-reactive | ATLG | 51242 | 1525 | 528 | 80 |
| R154 | 1M | ATLG | 538595 | 211018 | 79809 | 32310 |
| R154 | 3M | ATLG | 163607 | 89604 | 195946 | 45618 |
| R154 | 12M | ATLG | 225288 | 83706 | 96929 | 18979 |
| R192 | PreTX | ATLG | 75905 | 44220 | 68526 | 11936 |
| R192 | Donor-reactive | ATLG | 431019 | 5414 | 400612 | 1717 |
| R192 | 1M | ATLG | 435045 | 106739 | 398677 | 39931 |
| R192 | 3M | ATLG | 218252 | 57037 | 419517 | 26314 |
| R192 | 12M | ATLG | 260641 | 68373 | 167980 | 15122 |
| R200 | PreTX | ATLG | 333771 | 69110 | 261291 | 17272 |
| R200 | Donor-reactive | ATLG | 523475 | 4809 | 422165 | 3112 |
| R200 | 1M | ATLG | 179989 | 47444 | 47191 | 12649 |
| R200 | 3M | ATLG | 241581 | 65362 | 45651 | 13084 |
| R200 | 12M | ATLG | 196852 | 42969 | 55532 | 8977 |
| R294 | PreTX | ATLG | 72247 | 26328 | 145110 | 7184 |
| R294 | Donor-reactive | ATLG | 200420 | 3913 | 199112 | 662 |
| R294 | 1M | ATLG | 72920 | 28682 | 34192 | 6926 |
| R294 | 3M | ATLG | 110519 | 25486 | 65729 | 4731 |
| R294 | 12M | ATLG | 235475 | 48614 | 69875 | 5496 |
| R327 | PreTX | ATLG | 61574 | 45663 | 91715 | 18187 |
| R327 | Donor-reactive | ATLG | 121378 | 6218 | 161124 | 8841 |
| R327 | 1M | ATLG | 296136 | 83123 | 198689 | 31830 |
| R327 | 3M | ATLG | 198252 | 68936 | 143025 | 23882 |
| R327 | 12M | ATLG | 293341 | 82441 | 81018 | 15519 |

**Supplementary Table 5** Overview of unique clonotype and clone counts for each subject divided in CD4 and CD8 T-cells in the ATLG treatment group.

| **Patient** | **Timepoint** | **Group** | **Clones - CD4** | **Clonotypes - CD4** | | **Clones - CD8** | **Clonotypes - CD8** |
| --- | --- | --- | --- | --- | --- | --- | --- |
| R24 | PreTX | Basiliximab | 227949 | 60952 | | 170881 | 7373 |
| R24 | Donor-reactive | Basiliximab | 238455 | 12514 | | 102769 | 2711 |
| R24 | 1M | Basiliximab | 335480 | 83318 | | 280513 | 8742 |
| R24 | 3M | Basiliximab | 51438 | 25072 | | 33649 | 4025 |
| R24 | 12M | Basiliximab | 108943 | 41061 | | 136888 | 4018 |
| R30 | PreTX | Basiliximab | 327616 | 107735 | | 67913 | 11009 |
| R30 | Donor-reactive | Basiliximab | 1746446 | 156744 | | 95356 | 2091 |
| R30 | 1M | Basiliximab | 63989 | 36982 | | 27124 | 8605 |
| R30 | 3M | Basiliximab | 281059 | 86618 | | 72262 | 14165 |
| R30 | 12M | Basiliximab | 324840 | 126139 | | 70007 | 14842 |
| R190 | PreTX | Basiliximab | 58970 | 40638 | | 212468 | 87533 |
| R190 | Donor-reactive | Basiliximab | 1906491 | 76324 | | 1981948 | 28739 |
| R190 | 1M | Basiliximab | 95976 | 66323 | | 70079 | 38165 |
| R190 | 3M | Basiliximab | 264624 | 136654 | | 220665 | 80668 |
| R190 | 12M | Basiliximab | 115756 | 70186 | | 64910 | 27017 |
| R202 | PreTX | Basiliximab | 147146 | 76373 | | 51023 | 8332 |
| R202 | Donor-reactive | Basiliximab | 716071 | | 16448 | 29643 | 704 |
| R202 | 1M | Basiliximab | 479555 | | 134148 | 34338 | 8339 |
| R202 | 3M | Basiliximab | 112186 | | 40785 | 62652 | 5335 |
| R202 | 12M | Basiliximab | 171578 | | 72971 | 29479 | 7289 |
| R211 | PreTX | Basiliximab | 200433 | | 101255 | 216239 | 58325 |
| R211 | Donor-reactive | Basiliximab | 166468 | | 10832 | 243834 | 6820 |
| R211 | 1M | Basiliximab | 148772 | | 80576 | 67906 | 29420 |
| R211 | 3M | Basiliximab | 390861 | | 129817 | 196817 | 48097 |
| R211 | 12M | Basiliximab | 330320 | | 116464 | 269668 | 51696 |

**Supplementary Table 6** Overview of unique clonotype and clone counts for each subject divided in CD4 and CD8 T-cells in the basiliximab treatment group.

| **Sample** | **Group** | **CD4/CD8** | **PreTX** | **1M** | **3M** | **12M** |
| --- | --- | --- | --- | --- | --- | --- |
| R154 | ATLG | CD4 | 0.41 | 0.42 | 0.31 | 0.38 |
| R154 | ATLG | CD8 | 0.17 | 0.06 | 0.06 | 0.06 |
| R192 | ATLG | CD4 | 0.55 | 1.54 | 1.49 | 1.29 |
| R192 | ATLG | CD8 | 0.65 | 0.68 | 0.81 | 0.78 |
| R200 | ATLG | CD4 | 0.86 | 1.05 | 0.83 | 0.78 |
| R200 | ATLG | CD8 | 0.91 | 0.48 | 0.65 | 0.54 |
| R294 | ATLG | CD4 | 1.5 | 2.67 | 2.26 | 2.06 |
| R294 | ATLG | CD8 | 0.61 | 0.76 | 0.57 | 0.68 |
| R327 | ATLG | CD4 | 0.24 | 1.22 | 1.1 | 0.99 |
| R327 | ATLG | CD8 | 0.65 | 0.87 | 0.81 | 0.69 |
| R190 | Basiliximab | CD4 | 0.33 | 0.49 | 0.61 | 0.75 |
| R190 | Basiliximab | CD8 | 0.16 | 0.1 | 0.12 | 0.18 |
| R202 | Basiliximab | CD4 | 0.44 | 1.19 | 1.45 | 1.1 |
| R202 | Basiliximab | CD8 | 0.55 | 0.57 | 0.53 | 0.42 |
| R211 | Basiliximab | CD4 | 0.49 | 0.73 | 0.72 | 0.73 |
| R211 | Basiliximab | CD8 | 0.14 | 0.15 | 0.15 | 0.17 |
| R24 | Basiliximab | CD4 | 1.47 | 2.94 | 2.94 | 1.92 |
| R24 | Basiliximab | CD8 | 2.74 | 5.58 | 5.34 | 5.12 |
| R30 | Basiliximab | CD4 | 0.87 | 2.86 | 3.86 | 2.05 |
| R30 | Basiliximab | CD8 | 0.73 | 0.99 | 0.89 | 0.58 |

**Supplementary Table 7** Percentage of donor-reactive clonotypes detected in the bulk repertoires after normalization by downsampling to the smallest number of reads.


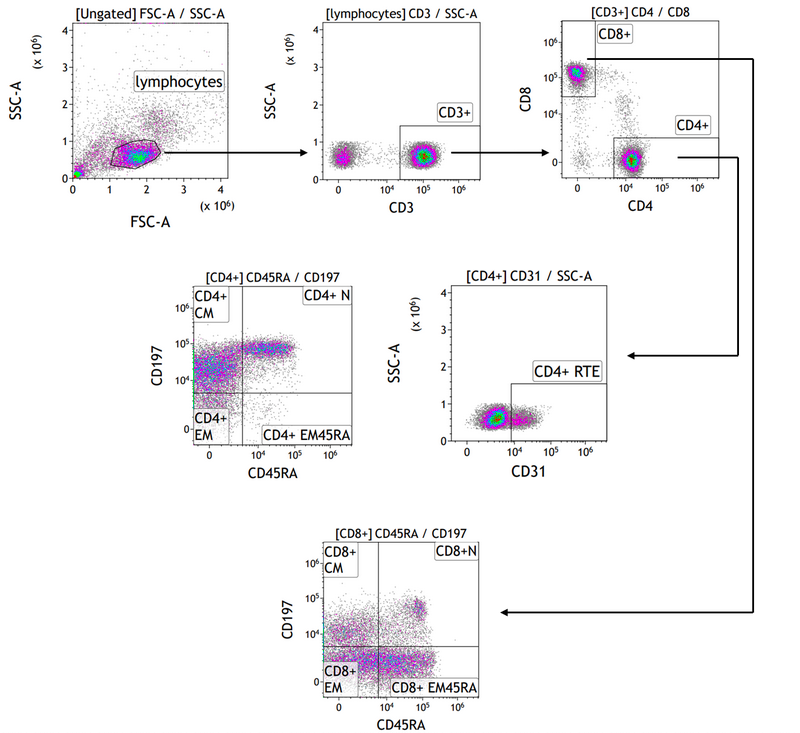


**Supplementary Figure 1** Representative FACS flow for phenotype analysis of reconstituted T-cells following reduced dose ATLG treatment.

**Supplementary Figure 2** Schematic overview of the method for identifying donor-reactive TCR’s. Irradiated VPD-labeled donor and CFSE-labeled recipient PBMC’s are incubated for six days in a MLR followed by FACS-sorting. Proliferating, CFSE-low CD4 and CD8 T-cells are sorted, followed by RNA isolation and TCR beta sequencing. CD4 and CD8 T-cells from the pre-transplant PBMC sample are also FACS sorted and TCR beta sequencing is performed after RNA isolation on these unstimulated cells.

| A)  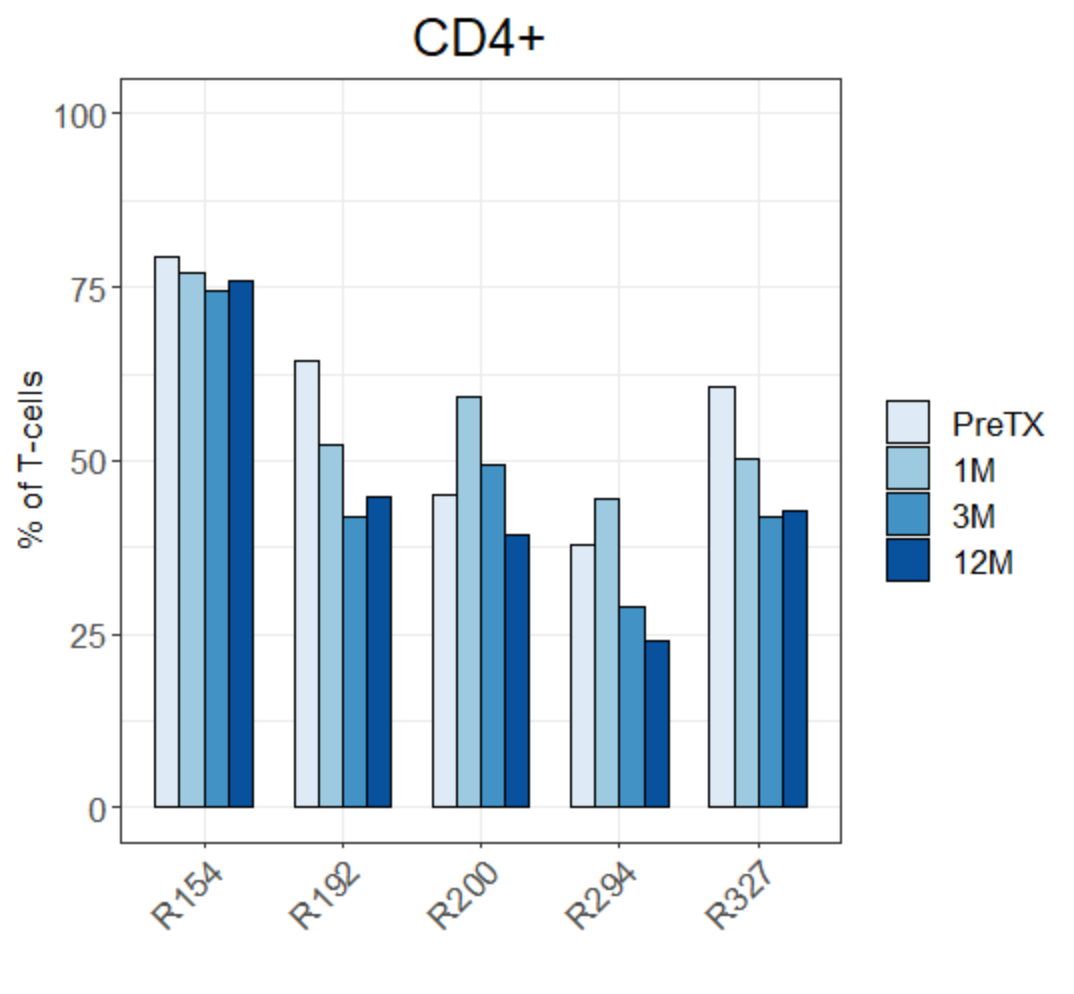 | B)  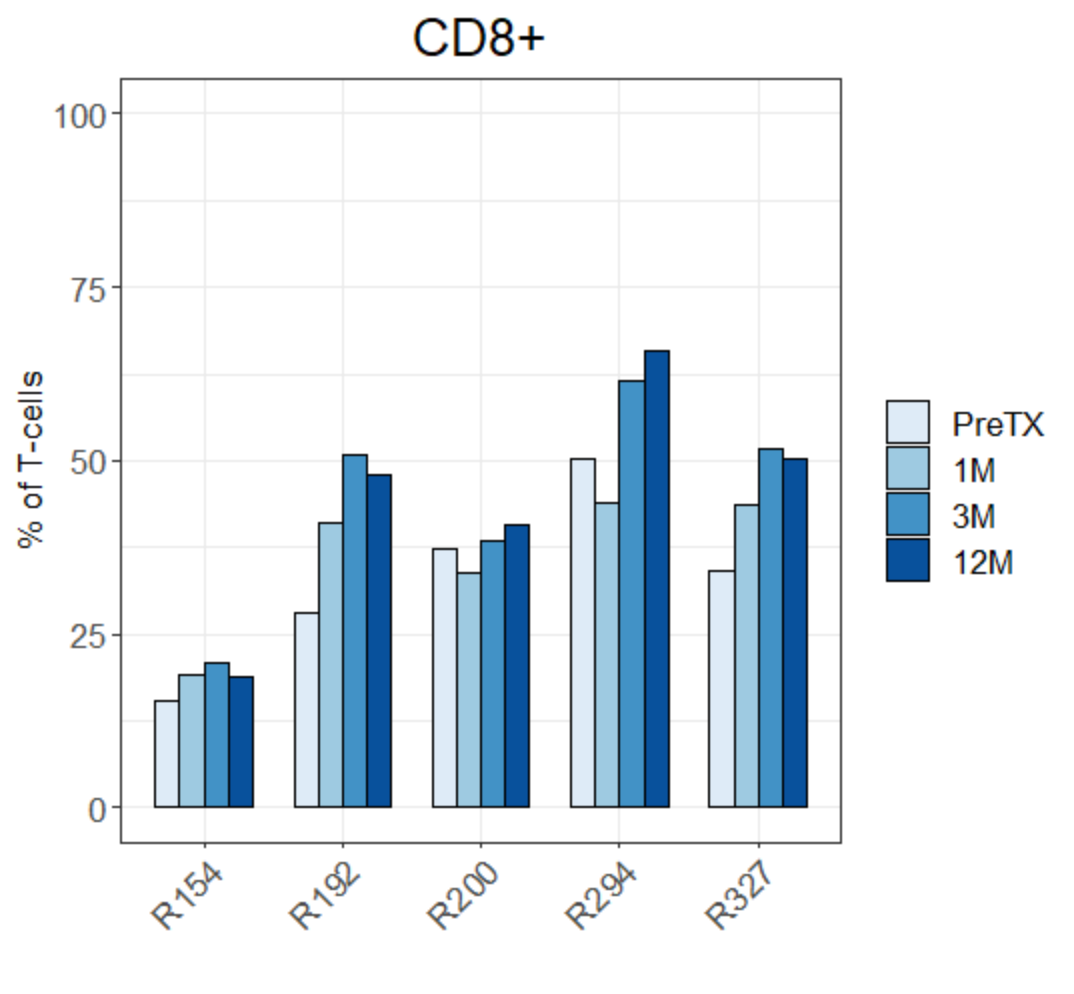 |
| --- | --- |
| C)  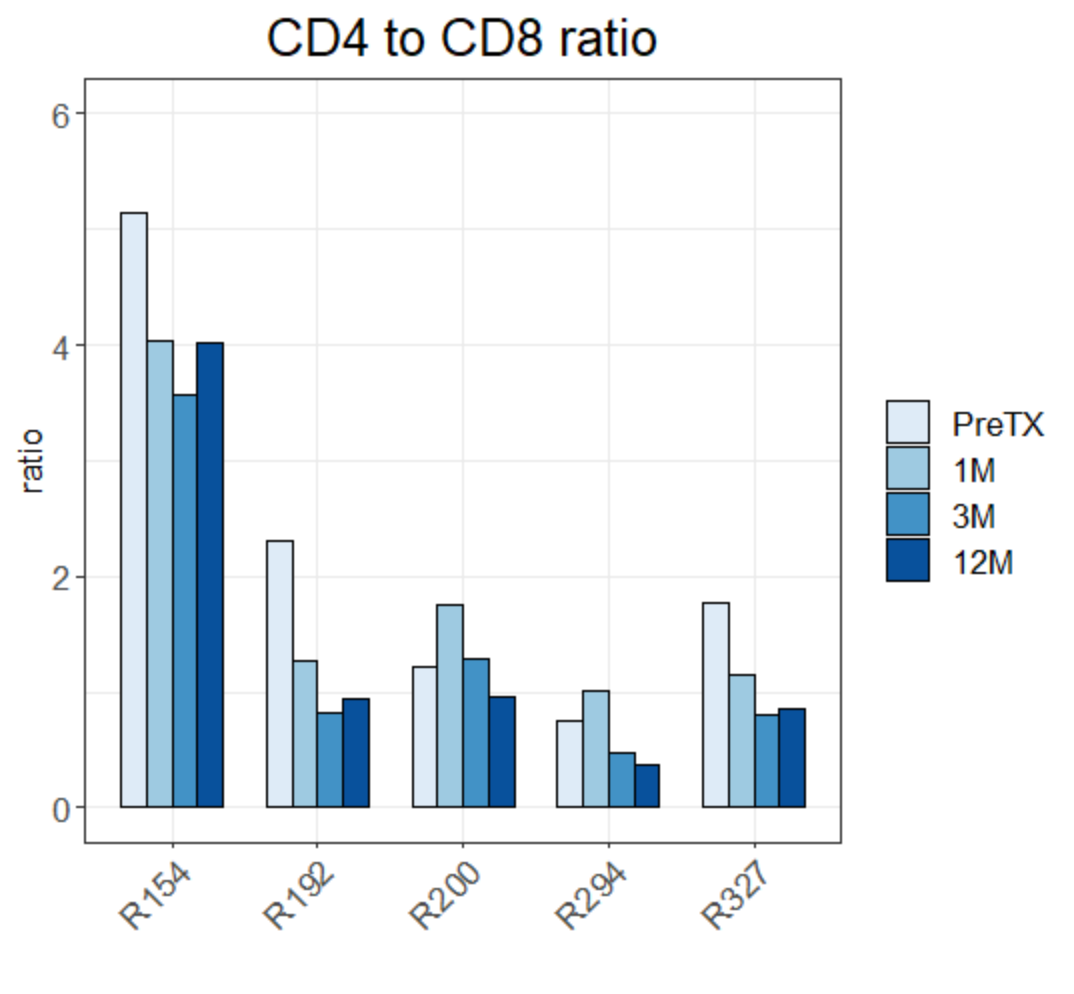 | |

**Supplementary Figure 3** CD4 and CD8 cells in the group of ATLG inducted patients throughout the study. Visualized are (A) percentage of CD4 T-cells among all T-cells, (B) percentage of CD8 T-cells among all T-cells and (C) ratios of CD4 to CD8 T-cells for each patient at each time point.

| A)  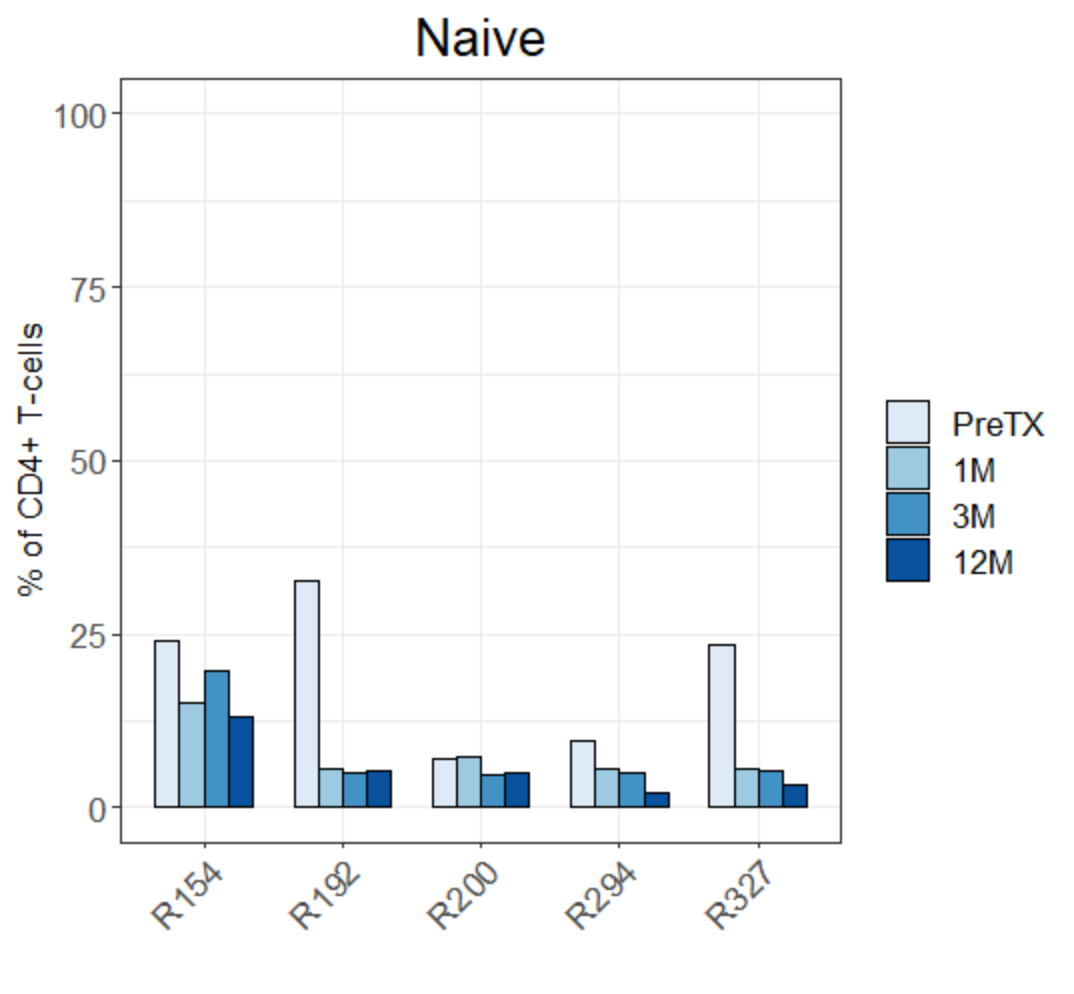 | B)  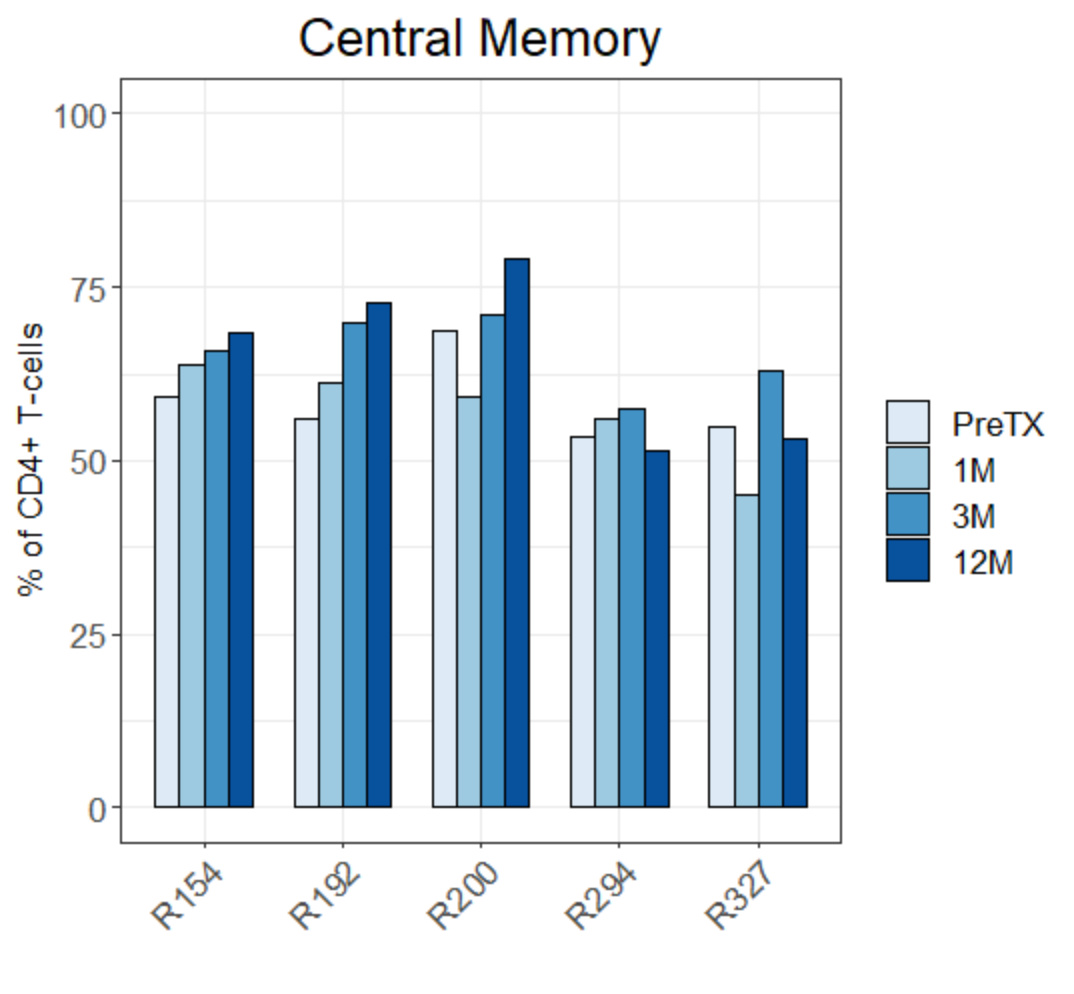 |
| --- | --- |
| C)  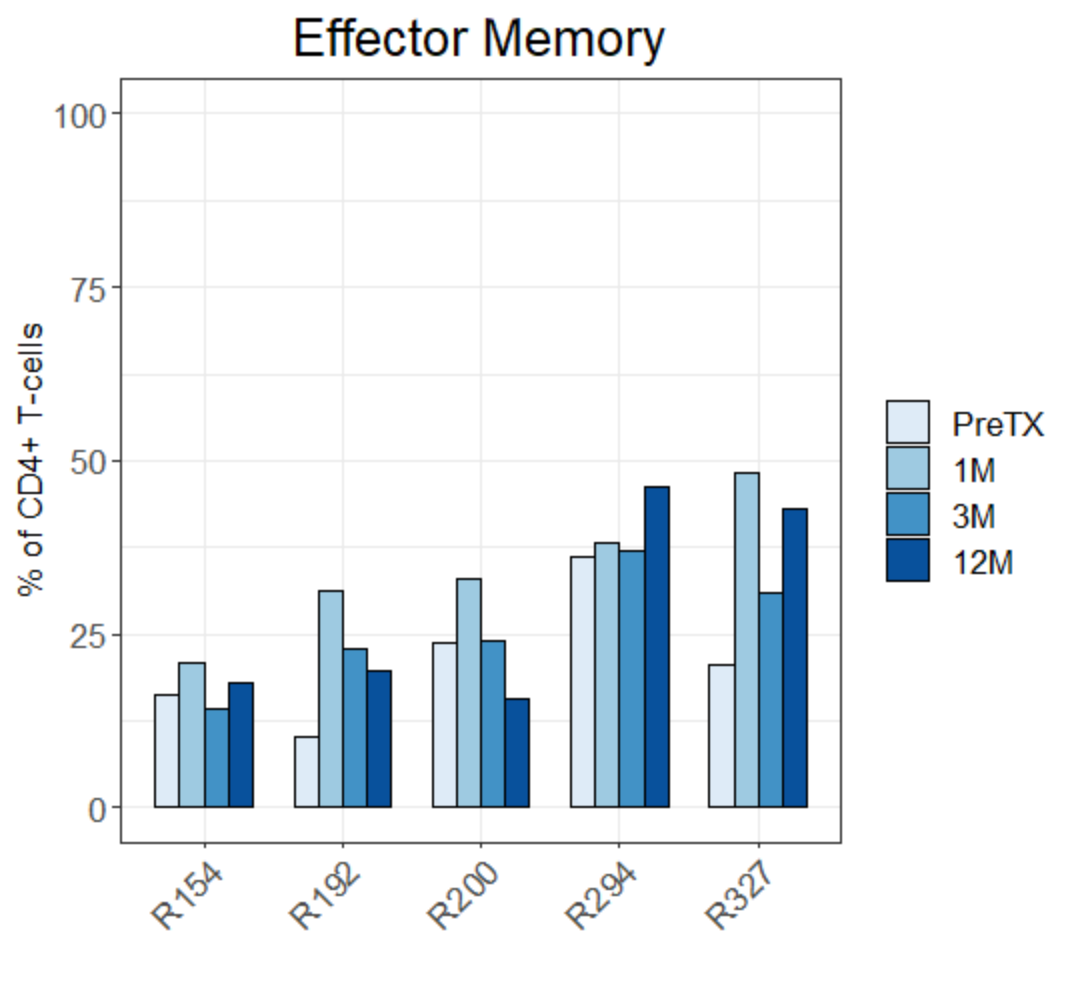 | D)  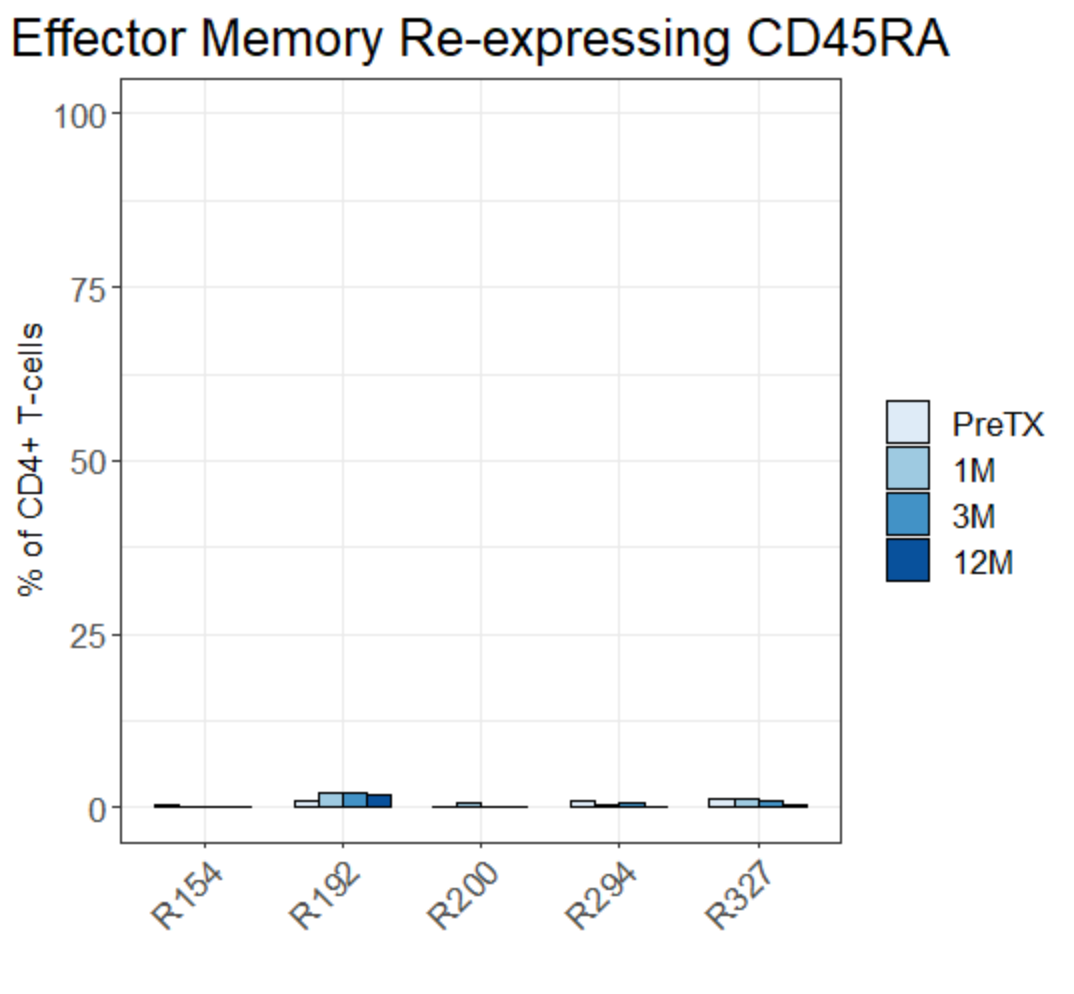 |

**Supplementary Figure 4** Naive and memory CD4 T-cells in the group of ATLG inducted patients throughout the study. Visualized are percentage of (A) naive T-cells (CCR7+, CD45RA+) among all CD4 T-cells, (B) central memory T-cells (CCR7+, CD45RA-) among all CD4 T-cells, (C) effector memory T-cells (CCR7-, CD45RA-) among all CD4 T-cells and (D) effector memory T-cell re-expressing CD45RA (CCR7-, CD45RA+) among all CD4 T-cells for each patient at each time point.

| A)  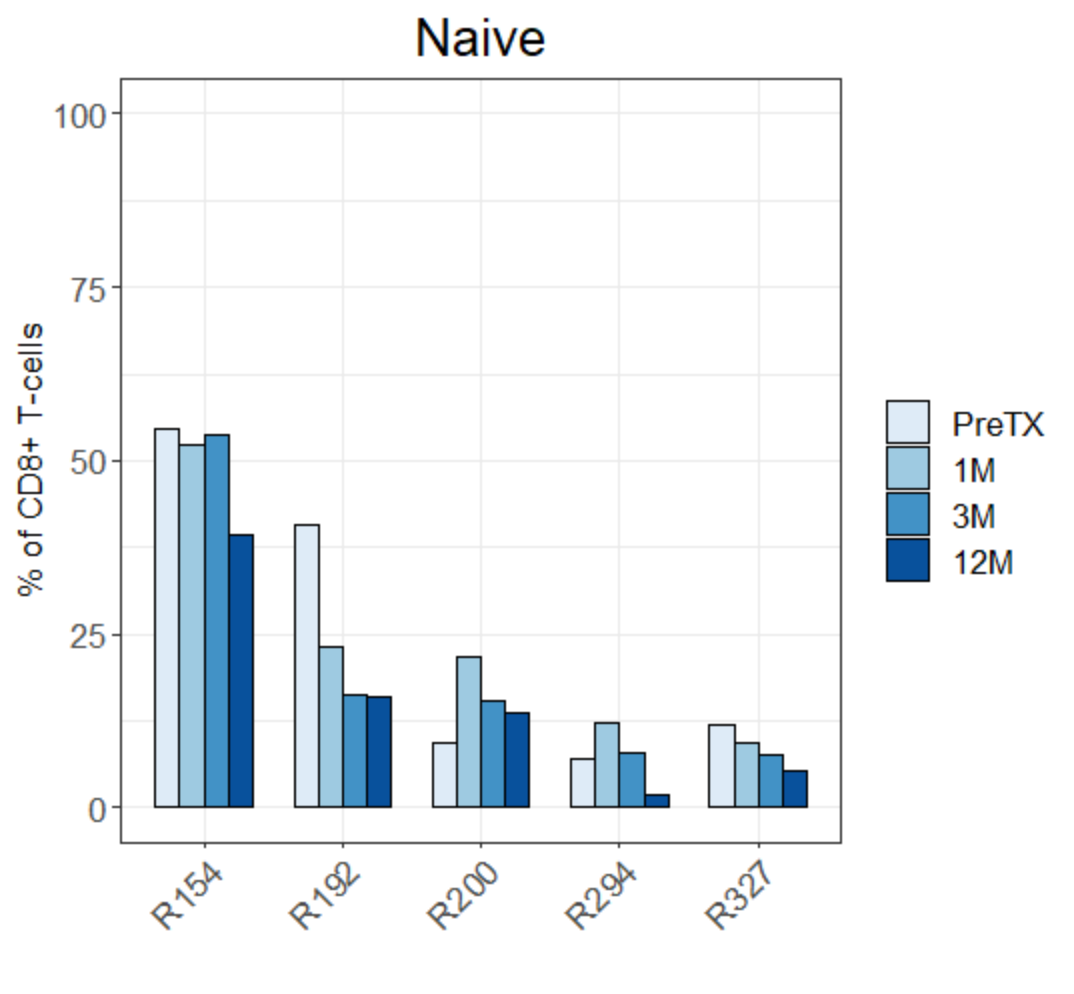 | B)  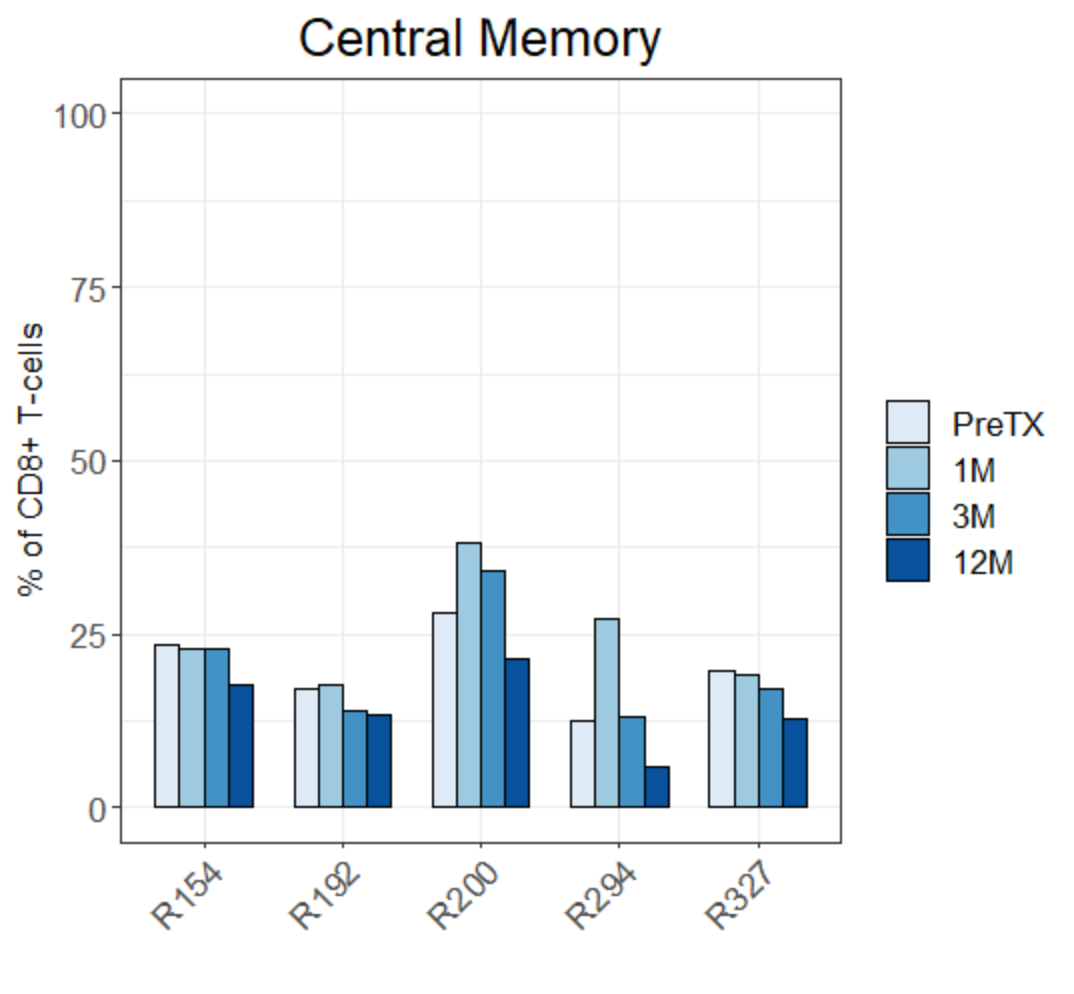 |
| --- | --- |
| C)  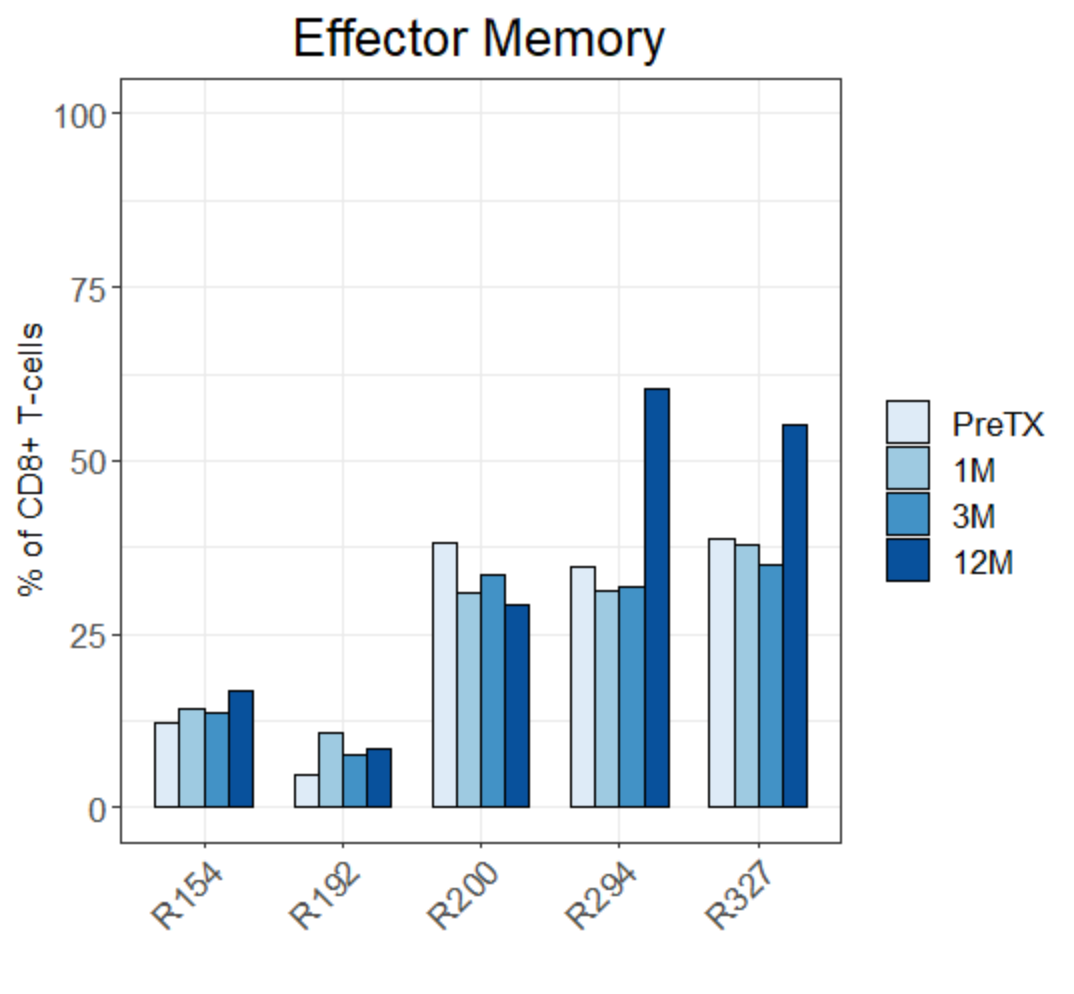 | D)  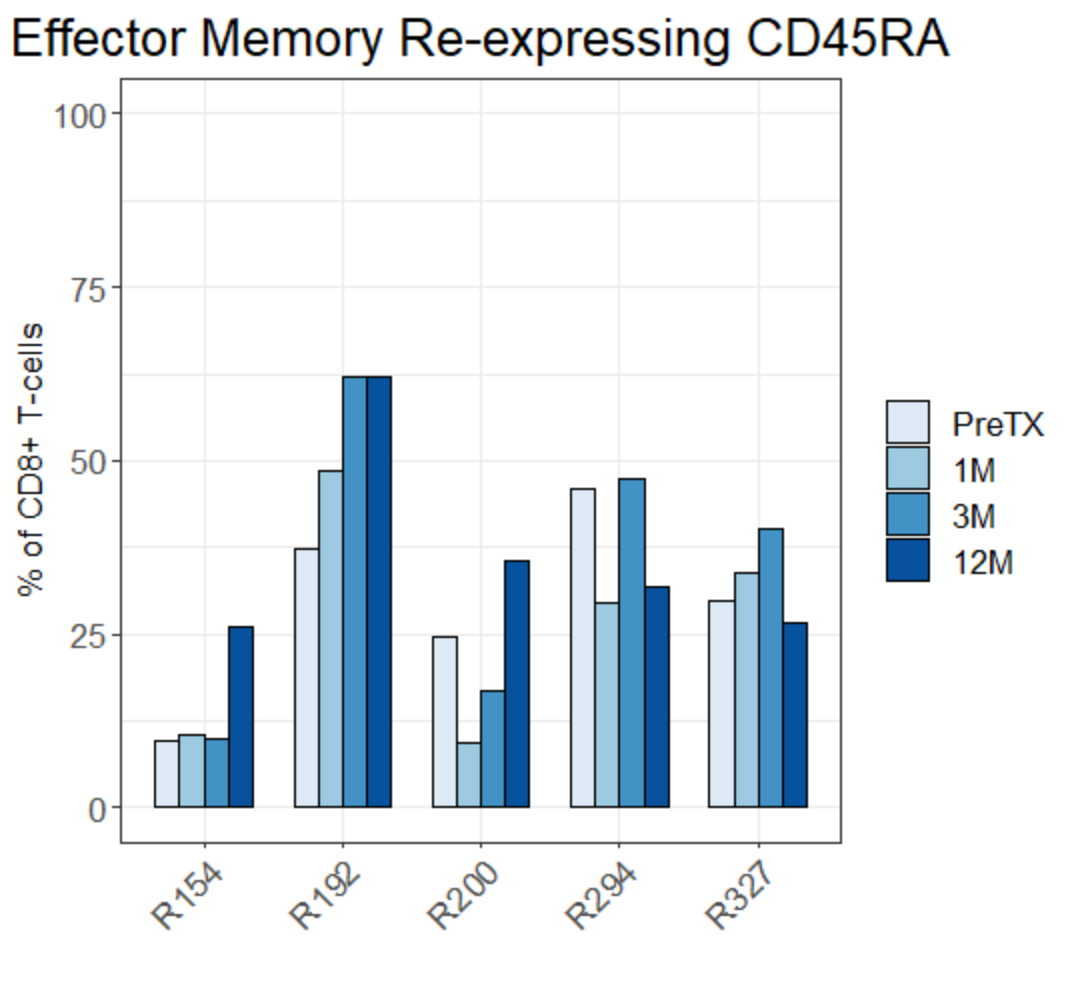 |

**Supplementary Figure 5** Naive and memory CD8 T-cells in the group of ATLG inducted patients throughout the study. Visualized are percentage of (A) naive T-cells (CCR7+, CD45RA+) among all CD8 T-cells, (B) central memory T-cells (CCR7+, CD45RA-) among all CD8 T-cells, (c) effector memory T-cells (CCR7-, CD45RA-) among all CD8 T-cells and (d) effector memory T-cells re-expressing CD45RA (CCR7-, CD45RA+) among all CD8 T-cells for each patient at each time point.


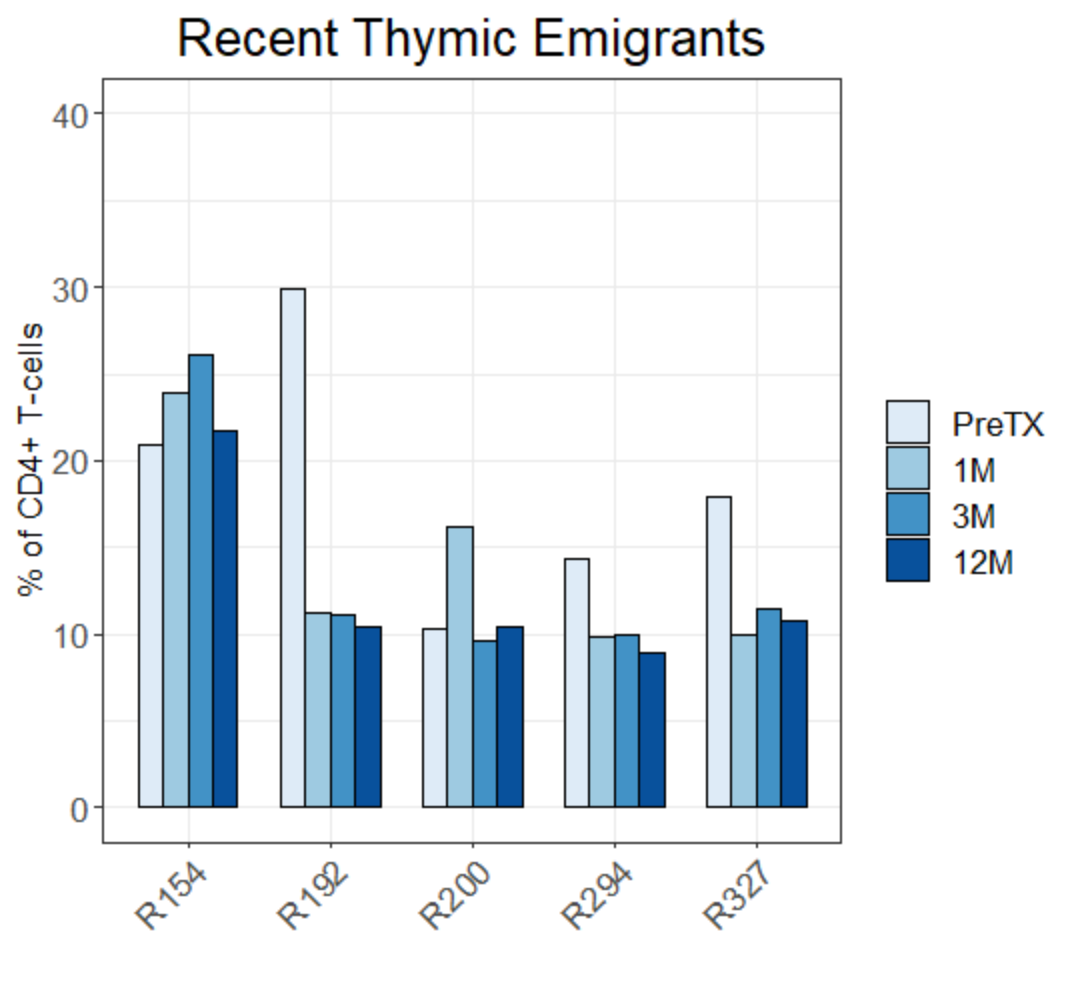


**Supplementary Figure 6** Recent thymic emigrants in the group of ATLG inducted patients throughout the study. Visualized is the percentage of recent thymic emigrants (CD31+) among all CD4 T-cells for each patient at each timepoint.

| A)  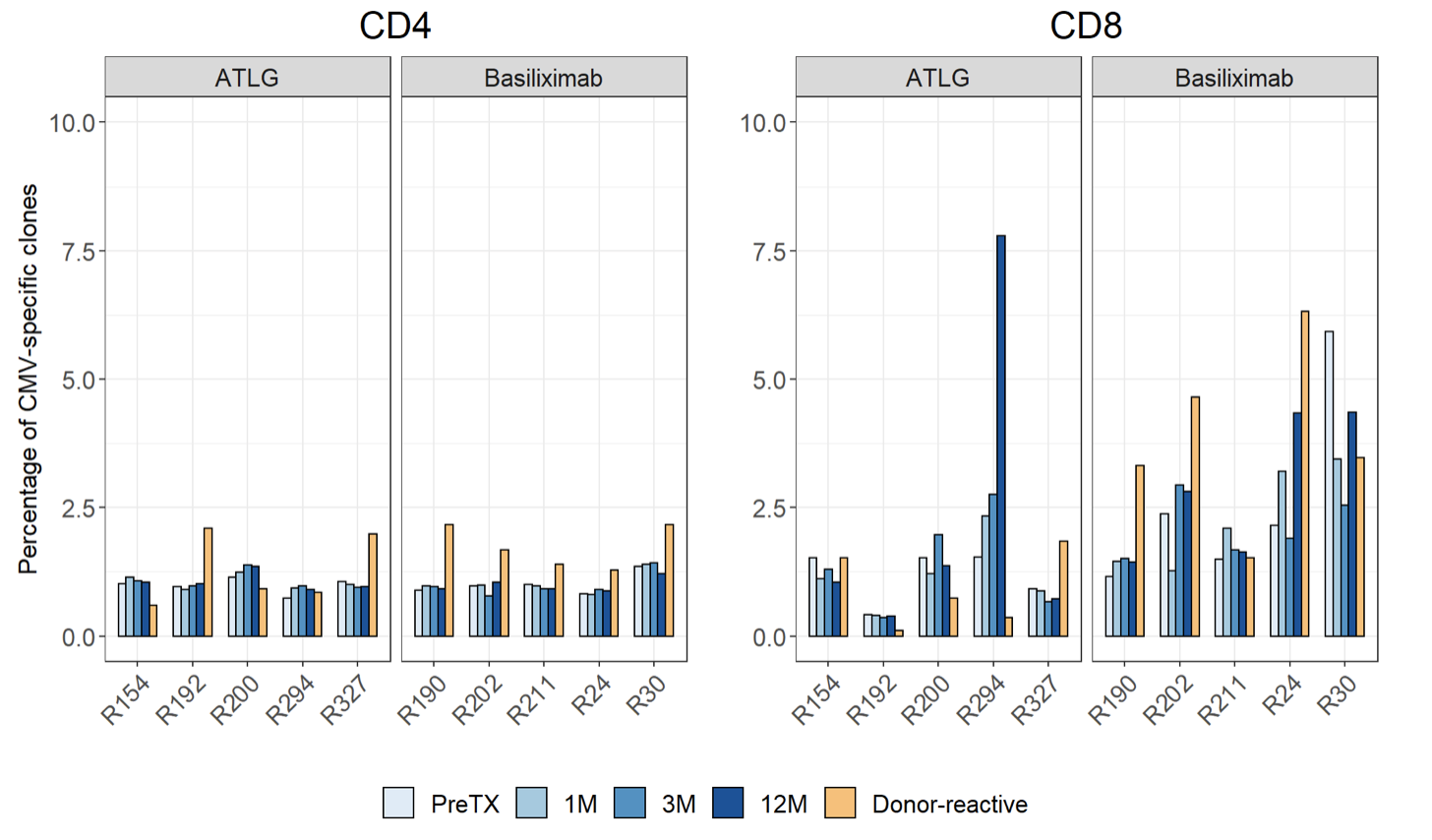 |
| --- |
| B)  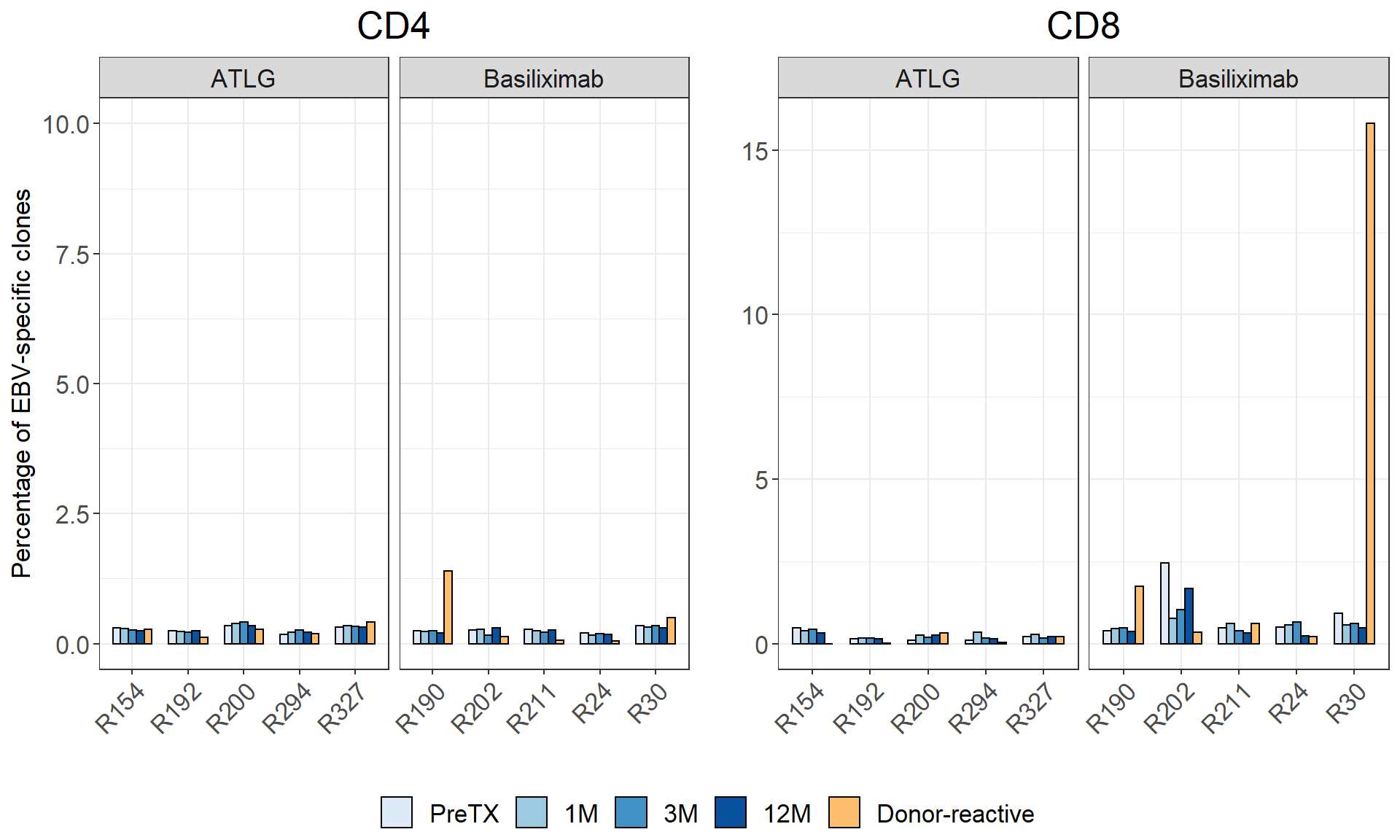 |

**Supplementary Figure 7** Percentage of (A) CMV and (B) EBV specific clones found in the TCR repertoires throughout the study. Virus specificity of clones was determined based on annotation available in the VDJdb database. The high percentage of EBV specific clones found in the donor-reactive TCR repertoire from R30 is driven by a single clonotype with the CDR3 amino acid sequence CASSARTGELFF.
